# The Smallest Known Genomes of Multicellular and Toxic Cyanobacteria: Comparison, Minimal Gene Sets for Linked Traits and the Evolutionary Implications

**DOI:** 10.1371/journal.pone.0009235

**Published:** 2010-02-16

**Authors:** Karina Stucken, Uwe John, Allan Cembella, Alejandro A. Murillo, Katia Soto-Liebe, Juan J. Fuentes-Valdés, Maik Friedel, Alvaro M. Plominsky, Mónica Vásquez, Gernot Glöckner

**Affiliations:** 1 Alfred Wegener Institute for Polar and Marine Research, Bremerhaven, Germany; 2 Department of Molecular Genetic and Microbiology, Faculty of Biological Sciences, Pontificia Universidad Católica de Chile, Santiago, Chile; 3 Millenium Nucleus EMBA, Santiago, Chile; 4 Leibniz Institute for Age Research-Fritz Lipmann Institute, Jena, Germany; 5 Institute for Biochemistry I, University of Cologne, Cologne, Germany; 6 Leibniz Institute for Freshwater Ecology and Inland Fisheries, Berlin, Germany; Institut Pasteur, France

## Abstract

Cyanobacterial morphology is diverse, ranging from unicellular spheres or rods to multicellular structures such as colonies and filaments. Multicellular species represent an evolutionary strategy to differentiate and compartmentalize certain metabolic functions for reproduction and nitrogen (N_2_) fixation into specialized cell types (e.g. akinetes, heterocysts and diazocytes). Only a few filamentous, differentiated cyanobacterial species, with genome sizes over 5 Mb, have been sequenced. We sequenced the genomes of two strains of closely related filamentous cyanobacterial species to yield further insights into the molecular basis of the traits of N_2_ fixation, filament formation and cell differentiation. *Cylindrospermopsis raciborskii* CS-505 is a cylindrospermopsin-producing strain from Australia, whereas *Raphidiopsis brookii* D9 from Brazil synthesizes neurotoxins associated with paralytic shellfish poisoning (PSP). Despite their different morphology, toxin composition and disjunct geographical distribution, these strains form a monophyletic group. With genome sizes of approximately 3.9 (CS-505) and 3.2 (D9) Mb, these are the smallest genomes described for free-living filamentous cyanobacteria. We observed remarkable gene order conservation (synteny) between these genomes despite the difference in repetitive element content, which accounts for most of the genome size difference between them. We show here that the strains share a specific set of 2539 genes with >90% average nucleotide identity. The fact that the CS-505 and D9 genomes are small and streamlined compared to those of other filamentous cyanobacterial species and the lack of the ability for heterocyst formation in strain D9 allowed us to define a core set of genes responsible for each trait in filamentous species. We presume that in strain D9 the ability to form proper heterocysts was secondarily lost together with N_2_ fixation capacity. Further comparisons to all available cyanobacterial genomes covering almost the entire evolutionary branch revealed a common minimal gene set for each of these cyanobacterial traits.

## Introduction

Cyanobacteria are among the most successful primary producing aquatic organisms, having populated the Earth for approximately 2.8 billion years [1]. Extant species are major (occasionally dominant) components of marine, brackish and freshwater environments, where they play crucial roles in global biological solar energy conversion and nitrogen (N_2_) fixation, but are also found in terrestrial ecosystems (in mats), and as extreme thermophiles in hot springs and polar ice. In high biomass concentration, cyanobacteria are responsible for noxious or harmful algal blooms (HABs), and this phenomenon is compounded by the fact that some cyanobacteria also produce potent cyanotoxins (microcystins, nodularins, saxitoxins, anatoxins, cylindrospermopsins, etc.), which have been classified according to their mode of action and effects on mammals [2].

Cyanobacteria have evolved alternative morphologies, including unicellular and diverse multicellular forms ranging from simple colonies to branched filaments. Phylogenetic analysis has suggested that cyanobacteria capable of cell differentiation are monophyletic [3]. Within this monophyletic group some cyanobacteria further evolved from filaments in which a small number of vegetative cells differentiated into either heterocysts or akinetes (resting stages). Nitrogen (N_2_) fixation, or diazotrophy, also appears to be monophyletic among cyanobacteria, although a polyphyletic origin has also been proposed [4], [5]. When mineral and organic nitrogen sources, such as nitrate or ammonium, are depleted from the growth medium, some filamentous cyanobacteria maintain photosynthetic activity (including O_2_ generation) in vegetative cells and differentiate heterocysts to provide an anoxic environment suitable for N_2_ fixation [6].

The proposed evolutionary sequence of heterocyst-forming filamentous cyanobacteria is still under debate. However, a likely scenario is that diazotrophy was first established in filamentous cyanobacteria (who acquired it either by horizontal gene transfer (HGT) or by vertical evolution of a not necessarily filamentous ancestor), and only after the establishment of diazotrophy, the capacity for heterocyst formation in filamentous diazotrophs developed [5].

Among filamentous cyanobacteria, the toxigenic species *Cylindrospermopsis raciborskii* is highly successful in freshwater environments. This species has been reported to be rapidly expanding worldwide, from tropical to temperate freshwater bodies [7]. *C. raciborskii* can also co-exist with morphotypes assigned to the closely related genus *Raphidiopsis* (also with toxin-producing members), which unlike *Cylindrospermopsis* does not develop heterocysts or fix N_2_ [8].

One remarkable characteristic of some cyanobacteria is their ability to form toxic blooms. Nevertheless, toxigenicity is not a ubiquitous feature at the generic level or even within a species; for example, both non-toxic and toxic strains of *Cylindrospermopsis* and *Raphidiopsis* have been isolated from natural populations. The genes responsible for toxin production are organized into clusters that might be subject to frequent HGT, a possible explanation for the evolution and biogeography both toxigenic and non-toxigenic strains within species or genera [9]. Among *C. raciborskii* strains, two totally different types of toxins may be produced: the hepatotoxin cylindrospermopsin (CYN), a tricyclic alkaloid inhibitor of protein synthesis [10], or neurotoxins associated with paralytic shellfish poisoning (PSP), specifically the tetrahydropurine saxitoxin (STX) and analogues [11]. Cylindrospermopsin is biosynthesized via combined polyketide synthase/nonribosomal peptide synthetase (PKS/NRPS) pathways [12], whereas cyanobacterial STX and its analogues are likely generated by a unique gene cluster recently described in *C. raciborskii* strain T3 [13] and also in a few other toxic species [14]. Toxic strains of *Raphidiopsis* are reported to produce CYN and/or deoxycylindrospermopsin (doCYN) [15], the bicyclic amine alkaloid anatoxin-a [16], which affects mammalian nicotinic acetylcholine receptors, or PSP toxins [17]. These cyanotoxin classes exhibit completely different mechanisms of action in mammalian systems [10], [18] and are also structurally dissimilar.

Cyanobacteria are of high ecological importance, and given their relatively small genome, they are an ideal target for genome sequencing and analysis with current genomic tools. Knowledge gained from such projects has yielded important insights into the evolution of photosynthesis [19], and adaptations of these microorganisms to the environment [20]. Nevertheless, to date, only 9 filamentous cyanobacteria have been either completely or partially sequenced.

**Figure 1:**
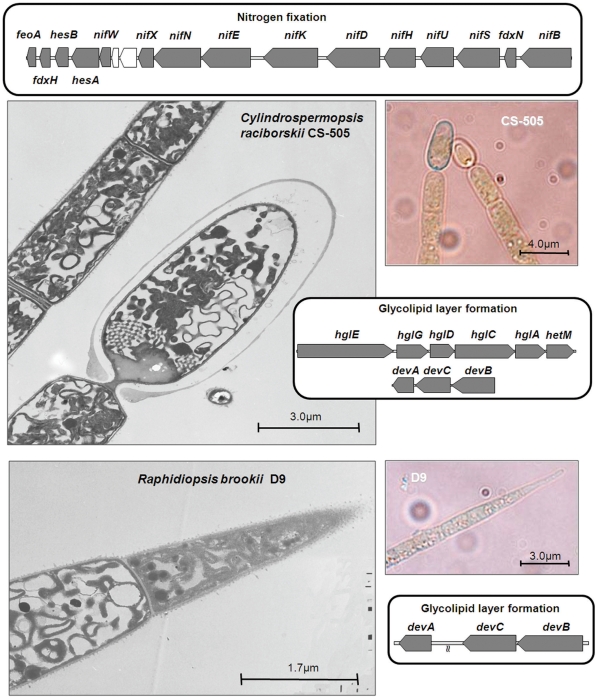
Overview of the main gene clusters involved in nitrogen metabolism and heterocyst development in strains CS-505 and D9. Transmission electron micrographs in the left panels show the heterocyst of CS-505 and the apically differentiated cell of D9. Optical micrographs on the right panels exhibit the Alcian blue staining characteristic of polysaccharides in the heterocyst.

Comparative genomics has also revealed high genetic variability even between closely related cyanobacterial strains [21]. Our objective was to conduct a genomic comparison of phylogenetically closely related filamentous cyanobacteria with a particular focus on the elucidation of the genetic background of their morphological and metabolic differences. Accordingly, we chose two cyanobacterial strains, *C. raciborskii* CS-505 and *R. brookii* D9 isolated from geographically disjunct regions in Australia (CS-505) and Brazil (D9) (Figure 1). The strains under study have been morphologically classified into different genera, since D9 produces no functional heterocysts and is therefore unable to fix N_2_. Nevertheless, based on 16S rDNA analysis they share 99.5% identity and are thus part of the same monophyletic cluster [22]. The two chosen strains also express a radically different toxin profile: while CS-505 produces exclusively CYN and doCYN, strain D9 produces the PSP-toxin analogues STX, gonyaulaxtoxins 2/3 (GTX2/3) and the respective decarbamoylated analogues [22].

We sequenced and analyzed the complete genomes of both strains CS-505 and D9, and thereby found the two smallest genomes thus far described for filamentous cyanobacteria. A comparative genomic analysis of these strains in relation to other members of filamentous cyanobacteria allowed us to propose minimal sets of core genes that provide insight into the evolution of diazotrophy and multicellularity, and heterocyst development in these minimal genomes.

## Results and Discussion

### Genome Structure Comparison

We sequenced the genomes to >20-fold depth with 454/Roche pyrosequencing technology (Table 1), thereby rendering >99.9% complete genomes [23]. Although a number of small gaps caused mainly by repetitive elements remain in both sequences, it is thus unlikely that we missed a significant portion of the genomes. The additional sequences of the long and short insert libraries from the Sanger sequencing (Table 1) also served to mitigate this deficiency, and the extra Sanger sequences derived from the short insert libraries were used to correct for 454/Roche technology intrinsic errors.

**Table 1:** Sequencing and assembly statistics for the two strains.

	D9	CS-505
454 GS sequence coverage	27	34
Small insert library	188	3909
Fosmid library	491	--
Finishing reads	253	161
Total sequencing depth	27	35
Contigs	157	268
Assembled (Mb)	3.20	3.89
Contigs >3.5 kb	33	94
Largest contig (kb)	543	259
Repeats (regions)	53	406
Repeats (bases)	53,870	244,280
Repeats (% total)	1.7	6.3

An initial assembly of all sequencing data for each strain yielded 182 contigs (larger than 3 kb) for CS-505 and 105 for D9. Due to limitations in the assembly of next generation sequencing (NGS)-derived, repeated sequences are commonly represented only once in such an assembly. Thus, only plasmid shotgun and fosmid clone end-sequencing and clone walking enabled us to close further gaps, such that the current assembly consists of 94 contigs for CS-505 and 33 for D9 (Table 1). The highly repetitive nature of the remaining gaps prevented us from reconstructing gap-free genomes.

Contigs lengths circumscribe an overall genome size of 3.89 Mb for strain CS-505 versus a smaller genome size of 3.20 Mb for D9 (Table 1); sequences in gaps accounted for an additional estimated 100 to 150 kb in both strains. The size of the genomes was further supported in the assessment by restriction fragment length polymorphism with Pulsed Field gel electrophoresis (PFGE) (Methods S1). The later method rendered an estimated genome size of 3.49 Mb for CS-505 and 3.09 Mb for D9 using the restriction enzyme *Mlu* I. The smaller estimated sizes by PFGE can be attributed to the low resolution of the some bands in the electrophoresis, which may lead to an underestimation of the genome sizes (Figure S1). Indicative of the presence of plasmids, we observed a faint band in CS-505 (Figure S2) plus a second band (data not shown) of approximately 30 kb. Plasmids may be integrated into the genome and thereby the plasmid sequences can be present in the assemblies surrounded by two different sequence environments (plasmid only sequences or adjacent genome parts), making the integration site a low coverage region. Thus, probably a plasmid is, if there is any, very likely represented by a single contig in our assembly.

The genomes we sequenced are almost a factor of two smaller than that of the most closely related fully sequenced cyanobacterium, *Anabaena* sp. PCC 7120 (hereafter referred as *Anabaena*) (6.41 Mb) (Table 2). The genome sizes of filamentous cyanobacterial species are previously reported to range between 5.0 and 8.7 Mb (NCBI database). Curiously, the genomes of our filamentous cyanobacteria are comparable to the genome size of those of unicellular cyanobacteria such as *Synechocystis* sp. PCC 6803 (3.57 Mb). Moreover, the number of ribosomal operons (3) and regulatory systems in both CS-505 and D9 (81 and 75 sensor-regulator components, respectively), is more similar to that of *Synechocystis* sp. PCC 6803 (3 ribosomal operons and 89 sensor-regulator systems), to that of the filamentous *Anabaena* (4 ribosomal operons and 175 sensor-regulator systems).

**Table 2:** General features of the genomes of strains CS-505 and D9 in comparison with four other fully sequenced genomes of filamentous cyanobacteria.

	D9	CS-505	Avar	Anab	Tery	Npun
Genome size (Mb)	3.20	3.89	6.34	6.41	7.75	8.23
G+C content (%)	40	40.2	41	41	40.8	41
Genes	3,088	3,968	5,134	5,432	5,542	6,501
Total CDS	3,010	3,452	5,039	5,368	4,452	6,087
Function assigned*	1,979	1,922	3,799	3,892	2,729	0
Unclassified	1,031	1,530	1,244	1,474	2,347	6,087
rRNA genes	9	9	12	12	5	12
tRNA genes	42	42	47	48	38	98
Transposases	9	77	57	145	260	112
Phage integrases	-	2	10	-	3	22
Repeated regions	53	406				
Plasmids	?	?	3	6	-	5
Unique CDS	394	794				
Function assigned*	157	291				
Unclassified	237	503				

Abbreviations: Avar: *Anabaena variabilis* ATCC 29413; Anab: *Anabaena* sp. PCC 7120; Tery: *Trichodesmium erythraeum* IMS101; Npun: *Nostoc punctiforme* PCC 73102.

*Function assigned according to COGs.

Genome reduction is a well-known evolutionary strategy to streamline genomes and get rid of superfluous functions. This strategy is followed by most obligate pathogens because their metabolic processes are strongly dependent upon the host. However, free-living cyanobacteria undergo genome reduction as well [20]. The reasons for this genome reduction phenomenon are unknown, but are likely related to genomic efficiency and relatively lax selective pressure on certain aspects of metabolism.

The G+C content is similar between both genomes (approximately 40%) and also similar to that of other fully sequenced genomes of filamentous cyanobacteria (Table 2). The genomes share 2539 clearly orthologous protein coding sequences (CDS) (referred to here as shared CDS), representing 73.6% and 84.4% of all predicted CDS from CS-505 and D9, respectively. We found 112 additional CDS in the CS-505 genome with similarities to D9 counterparts. Further analysis indicated that this surplus of CDS is mainly due to coding parts of transposable elements (data not shown, but note following discussion). Of the shared genes, the average nucleotide identity is >90% and the rate of synonymous substitutions is 0.29. These values are similar to those found for conspecific bacterial strains that have evolved in different ecological habitats [24] and are consistent with the level of similarity between 16S rRNA sequences from CS-505 and D9.

### Unique CDS in the Genomes

Comparative analysis via Best Bidirectional Hits (BBH) revealed large differences in the number of unique CDS between the two strains. CS-505 has 794 (23%) unique CDS whereas D9 contains 394 (13%). The presence and number of unique CDS among two closely related strains may represent the different potential for ecological adaptation and physiological plasticity. This relationship has been proven, particularly for pathogenic bacterial isolates that acquire pathogenicity islands conferring the toxic phenotype [25]. Even the acquisition of single genes can yield adaptations to a specific strain. For example, the two ecotypes of *Prochlorococcus marinus* MIT9313 and MED4 differ in the presence of certain Photosystem II and nitrite transport and reduction genes, among others. These differences correlate with the distribution of the ecotypes in the water column [20].

**Figure 2:**
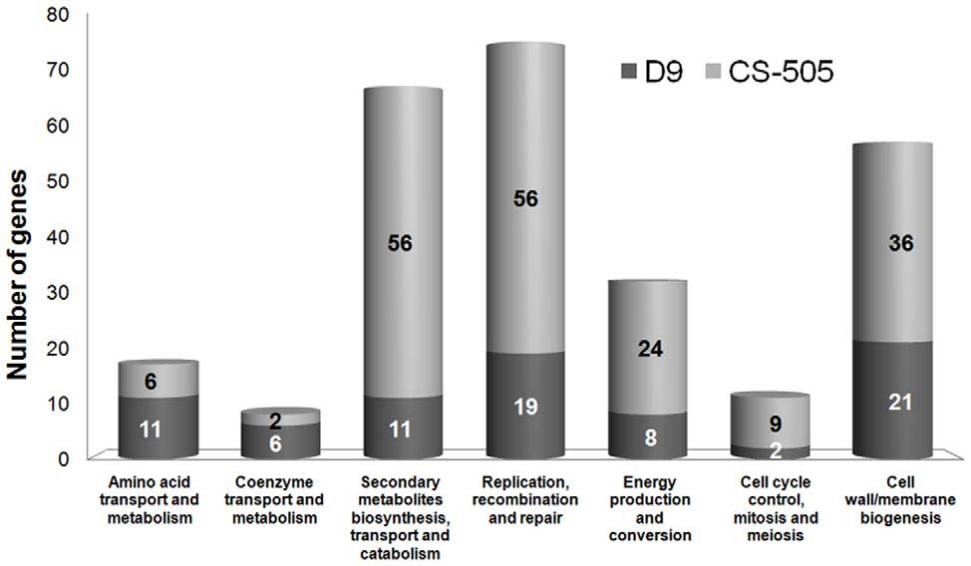
Distribution of the unique CDS of CS-505 and D9 into Cluster of Orthologous Groups (COGs). Only COG categories overrepresented by CDS of CS-505 or D9 are shown (see text for more details). Unique CDS were obtained by a Best-Bidirectional Hits (BBHs) search between both genomes using a 30% cutoff.

The classification of the unique CDS into Clusters of Orthologous Groups (COGs) showed that for two thirds of the unique CDS no function could be assigned (503 of 794 for CS-505 and 237 of 394 for D9). Yet of the remainder, there was a homogeneous distribution within most of the COG categories, indicating common functions between CS-505 and D9 (Figure S3, Table S1). Only a minor fraction of the unique CDS of both strains showed evident differences in their distribution into the different categories. Those differences were restricted to seven COGs (Figure 2) from which only two categories were better represented by D9-unique CDS: coenzyme and amino acid transport and metabolism. The COG distribution clearly showed the greater metabolic capabilities of CS-505 than D9 in relation to: 1) secondary metabolite biosynthesis, transport and catabolism; 2) replication, recombination and repair (this category is overrepresented partially due to transposons), 3) energy production and conversion, 4) cell cycle control and 5) cell wall and membrane biogenesis. On closer inspection, most of the identifiable unique CDS of CS-505 were organized into gene clusters and could be attributed to toxin production and heterocyst differentiation coupled with diazotrophy (discussed in more detail below). Thus, the lack of these genes and the scarcity of unique genes in D9 points to the fact that this genome was shaped by gene and function losses rather than gains.

### Repetitive Elements and Synteny

The most prominent difference between the genomes of CS-505 and D9 is the overwhelming number of repeated insertion elements or transposon-derived sequences in the CS-505 genome, which accounts for a considerable part of the genome size difference (nearly 0.6 Mb or 20% of the D9 genome) between the two strains (Table 1). Repetitive elements are not rare in cyanobacteria. On the contrary, a high percentage of repeated sequences was found in the genomes of *Crocosphaera watsonii* WH8501 (19.8%) and in the only two sequenced *Microcystis aeruginosa* strains (11.7% each) [26], [27]. However, our study represents the first time that large differences in repeat numbers have been observed between closely related strains. A low number of CDS (∼100) in the CS-505 genome reflects apparent gene duplications or functional redundancies. However, since we produced significantly more large- and small-insert library derived reads for the CS-505 strain from Sanger-based sequencing, a small portion of the observed genome size difference could be due to the better resolution of repeats in this strain. Such redundancy of long stretches of nearly identical sequences also contributes to our difficulties in closing the gaps in the genome sequences, particularly for CS-505. The total number of nearly identical repeated sequences with coding potential in the CS-505 genome accounts for 6.3% of its genome length. In addition, we identified two phage integrase genes and 77 transposases among them, from which only 28 were full sequences reflecting possible functionality. The low number of mobile elements in the D9 genome compared to other cyanobacteria is indeed remarkable. The presence of only 9 transposases (just one is a full sequence), 53 repeated regions and no phage integrase genes points to a high plasticity of the CS-505 genome relative to the transposon-poor genome of D9 (Table 2). Pursuit of repeated sequence elements not necessarily coding for proteins, employing a strategy described in Abouelhoda *et al*., [28] (see methods), allowed us to define 20 clusters that are present more than once in the CS-505 genome. Interestingly, one of those clusters internally repetitive, i.e. a short sequence stretch is repeated identically within this cluster several times (Figure S4) and occurs 39 times in the genome.

Repetitive elements can be a source for genome rearrangements. This genomic plasticity could be partly responsible for niche adaptation of organisms to their environment. We counted the number of syntenic regions between the two strains to estimate the number of rearrangements that occurred after their evolutionary separation. Interestingly, all 2539 orthologous gene pairs are located in syntenic regions, meaning that at least one neighboring CDS is common between the two strains. This excludes the possibility that single genes were relocated to other genomic regions during evolution. In total we found 280 synteny groups with a mean of 9 members in a group. The largest group comprised the ribosomal cluster and an adjacent CDS with 55 orthologous pairs. If we compare the *A. variabilis* genome to that of D9 in the same way we observe 464 synteny groups with only 1651 members. Thus, not unexpectedly, the mean of 3.6 CDS per synteny group is much lower than in the CS-505/D9 comparison. The high sequence similarity between CS-505 and D9 emphasizes the close relationship between the two strains, whereas the synteny analysis shows that rearrangements occurred relatively frequently during evolution. This high plasticity may be partly due to the high number of repeated elements.

### Genomic Islands for N_2_ Fixation and Toxin Production

**Figure 3:**
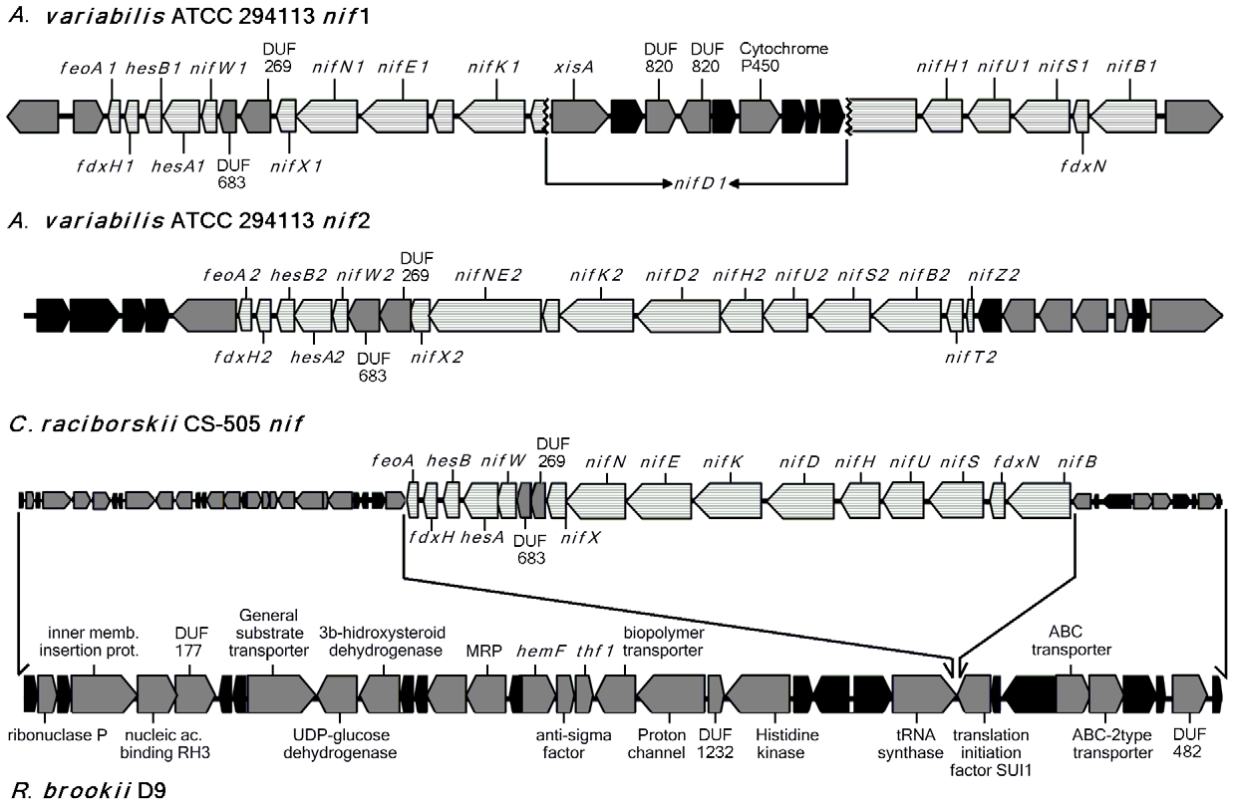
Schematic representation of the synteny within the vicinity of the *nif* gene clusters. The scheme represents the 15 kb gene cluster containing the *nifHDK* and the other 13 nitrogen fixation related genes in CS-505 compared with the *nif*1 and *nif*2 gene clusters of *Anabaena variabilis* ATCC 29413 and the synteny regions between CS-505 and D9. The synteny regions between CS-505 and D9 are delimited by the arrows. *nif* genes are represented by light grey and dashed lines. Genes in black correspond to hypothetical proteins and grey genes to proteins with assigned function.

We did not find any region matching the criteria for definition of a genomic island, i.e. differing G+C content, presence of direct repeats, transposition elements or tRNA sequences [25] within the CS-505 and D9 genomes. Nevertheless, we found gene clusters present in one or the other strain; thus we cannot discard the possibility that islands containing those gene clusters were transferred from genomes with a similar G+C content. Filamentous cyanobacteria are known to have a homogenous G+C content (Table 2). The most prominent examples of such identifiable gene clusters in our strains are those for N_2_ fixation and toxin production in CS-505, and the toxin production gene cluster in D9. In strain CS-505 the *nif* gene cluster encoding for the Fe-Mo cofactor-dependent nitrogenase and thirteen other genes related to N_2_ fixation are all together within a tight 15 kb cluster. The gene content is therefore similar to the *nif* cluster of heterocystous cyanobacteria [6]. The gene organization, however, is comparable to that of the second *nif* cluster expressed in vegetative cells of *Anabaena variabilis* [29], and of the *nif* cluster of the symbiotic *Nostoc azollae* 078 (see Figure 3 for the comparison with *A. variabilis*). The distinguishing feature of this gene organization is that it does not exhibit excision elements interrupting the *nifD* sequence, a characteristic of many other heterocyst-forming cyanobacteria. A second nitrogenase operon *nifVZT* (also commonly present in diazotrophic cyanobacteria) is located at a different locus in CS-505. The D9 strain is not able to fix N_2_ and is therefore dependent on the uptake of N-containing compounds from the environment. This dependency is nicely reflected by the absence of the N_2_-fixation gene clusters (*nif*) and the prevalence of several unique CDS for coenzyme- and amino acid- transport in the D9 genome (Figure 2). We note as significant that there is shared synteny in the regions surrounding the *nif* clusters in the compared strains (Figure 3). The *nif* clusters in *R. brookii* D9 might thus have been selectively lost along with the corresponding function. Nevertheless, the D9 genome encodes and expresses (Methods S1) *hetR*, an important regulator of heterocyst differentiation and pattern formation in N-fixing cyanobacteria [30], under normal culture conditions (with nitrate as N-source). As reported by Zhang *et al*., the presence of *hetR* and its expression have been detected in non-heterocyst producing cyanobacteria that also do not fix N_2_, pointing to a more global role of HetR [31].

**Figure 4:**
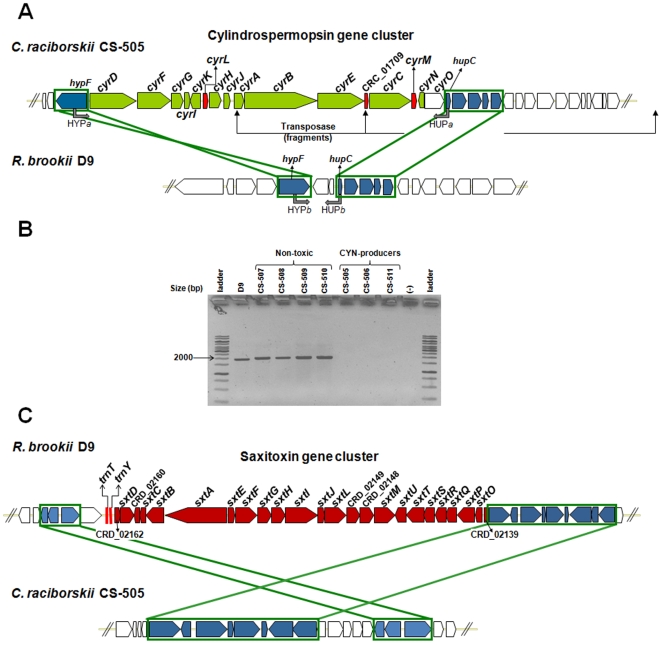
Schematic representation of the syntenic regions within the toxin gene clusters in CS-505 and D9. A. Location of the CYN gene cluster of CS-505 compared with the syntenic genomic region in D9. B. Gel electrophoresis of the PCR products from the *hypF/hupC* amplification in *R. brookii* D9 and in the strains of *C. raciborskii* non-toxic: CS-507, CS-508, CS-509 and CS-510. Producers of CYN: CS-505, CS-506 and CS-511 do not present amplification of the *hypF/hupC* region. C. Location of the STX gene cluster of D9 compared with the syntenic genomic region in CS-505. Genes participating in syntenic regions are depicted in blue and highlighted in the green boxes within the arrows; genes outside the syntenic regions are depicted in white. tRNAs and transposases are shown in red. The grey arrows show the position of the primer pairs HYPa/HUPa and HYPb/HUPb used to amplify the region between *hypF* and *hupC* genes in different strains of *C. raciborskii* and in *R. brookii* D9, respectively. Ladder: GeneRuler 1 kb DNA ladder (Fermentas, Ontario, Canada). The strains of *C. raciborskii* were obtained from the culture collection of the Commonwealth Scientific and Industrial Research Organization (CSIRO), Australia. For more details on DNA isolation, primer synthesis and PCR conditions see Methods S1.

A similar example of common and conservative elements is observed in the toxin gene clusters of CS-505 and D9. The different cyanotoxins produced by strains CS-505 and D9 are the most prominent known secondary metabolites in these cyanobacteria. The tricyclic alkaloids CYN/doCYN and the tetrahydropurine STX and analogues are N-rich molecules, but these toxin groups are synthesized by two independent and apparently unrelated biosynthetic pathways in cyanobacteria. The *C. raciborskii* CS-505 genome encodes for only one hybrid NRPS-PKS pathway, corresponding to the CYN/doCYN biosynthesis cluster. The cluster spans 41.6 Kb, encodes for 16 Open Reading Frames (ORFs) and has complete synteny with the CYN cluster of *C. raciborskii* AWT205 [12] (Figure S5A), flanked at both ends by genes from the hydrogenase gene cluster (*hypABCDEF*). In addition to the two transposase ORFs, *cyrL* and *cyrM*, CRC_01709 is only present in CS-505. This latter gene fragment of 219 bp is located between *cyrC* and *cyrE*, and matches with part of a transposase from *Synechococcus* BO 8402. The *cyrM* and CRC_01709 components are only vestiges of transposases, indicating that rearrangements have occurred in this section of the genome. The same genetic structure neighboring the CYN biosynthetic gene cluster in CS-505 is present in the strain D9, as another example of synteny (Figure 4A). As this conservation has been shown in other non-CYN producing Australian strains of *C. raciborskii* that contain the uninterrupted hydrogenase cluster (Figure 4B), we find it plausible that each cluster could be inserted or deleted at common genetic loci.

Likewise, the genes adjacent to the STX gene cluster in the D9 genome form a syntenic region within the CS-505 genome (Figure 4C). The STX gene cluster in D9 covers 25.7 Kb, and encodes for 24 ORFs, in comparison with 35 Kb and 31 ORFs described in the published STX gene cluster of *C. raciborskii* T3 [13] (Figure S5B). Only 20 ORFs are shared between these clusters (19 ORFs share 100% similarity); among these ORFs are all of the proposed genes necessary to synthesize STX. Thus, according to the genome size of D9, this is the minimum gene cluster thus far described for STX production.

### Tracing the Evolution of Traits in Cyanobacteria

Access to the smallest known genomes of filamentous and heterocyst-forming cyanobacteria provided insights into the molecular basis and evolution of traits such as diazotrophy, filamentous growth, and the capacity for cellular differentiation. We assumed that protein sequences had to be drastically changed or newly developed to achieve new functions. Of course it is possible that only slight neofunctionalizations could be responsible for the observed phenotypic changes without major restructuring. In the latter case, the observable gene repertoire of all cyanobacteria would remain relatively stable with only the addition of paralogous genes with acquired new functions. Genes with new functions would turn up at specific evolutionary time points and then remain stable as long as the respective trait is expressed and positively selected. Our analysis by definition excluded genes that might have become indispensible over the time course of evolution in one or another species, but not in all species analyzed. We thus aimed at only the description of key innovations for the establishment of major evolutionary branches.

To this end, we collected available genomes of cyanobacteria from the databases and compared their gene repertoire. Unfortunately, due to difficulties in culturing, no genome of a branching filamentous species (e.g. from Stigonematales) is available for comparison [32], but all other major groups are well represented (see Materials and Methods). The availability of streamlined genomes of *C. raciborskii* and *R*. *brookii* further enhanced the resolution of our analysis. We made use of the whole genome sequences and subtracted step-wise common sets from the sets represented only in species with specific traits. We cannot exclude that some genes in these sets are not related with the specific trait, but the broader the species sampling the better is the resolution of the genes of the trait in question. Since our analysis is based on BLAST hits, the orthologous relationships between the genes in the respective species may not be clear. But as it turned out later, most genes in the gene sets had only one counterpart in each genome, thus representing most likely orthologous gene groups. Table 3 shows a summary of the number of core genes found for each of the three traits under study.

**Table 3:** Common genes for the different traits.

Trait	Hits between species* (see methods)	CS-505	D9	Core set present in wider spectrum of species
Filament formation	32	23	20	10
Diazotrophy	49	38	6	10
Heterocyst development	149	58	54	41

*Paralogs are not removed.

### Filament Formation

Filaments are formed in groups III, IV and V of cyanobacteria [3]. Filament formation is also observed in unicellular cyanobacteria as well as in bacteria when several genes involved in cell division are interrupted by transposon mutagenesis [33]. If filament formation is generally a loss-of-function mutation then filamentous species should lack some cell division genes. However, orthologs of all genes examined for their effect on this artificial filament formation are present in the filament-forming species *Anabaena* (Table S2). Filament formation is thus more likely a gain-of-function in the evolutionary context. When we compared all the available genomes of filamentous species, we found 32 genes present in all (Table 3, Table 4). Comparison of this set with the more streamlined genomes of CS-505 and D9 showed that only 23 and 20 genes are present, respectively. Since the D9 strain is able to form proper filaments, the additional three genes found in the CS-505 genome are unlikely to be directly involved in filament formation. The absence of these three genes in D9 points to the probability that some of the remaining 20 genes are also not associated with ability to form filaments. This is further underlined by the fact that the additional screening of the unfinished genome sequences of *Nostoc azollae* 078 and *Microcoleus chtonoplastes* PCC 7420 yielded a common set of only 10 genes. We conclude that filament formation in cyanobacteria needs at most 10 different gene products. Interestingly, besides the three genes previously thought to be associated with heterocyst formation (*hetR* and *patU3* and *hetZ*) all other seven genes correspond to only hypothetical proteins. Although mutations in these three genes do not produce a unicellular phenotype, it has been shown that they affect heterocyst development, and *hetZ* and *patU3* also affect pattern formation [34], [35]. Their presence in cyanobacteria that do not present these phenotypes is suggestive of a different and more general function, which could be filament formation.

**Table 4:** Genes present only in filamentous species.

Npun		Gene product description	Anab	D9	CS-505
186680616		hypothetical protein	all1770	CRD_00231	CRC_00822
186680621	core set	hypothetical protein	all1765	CRD_00230	CRC_00821
186681198	core set	hypothetical protein	alr0202	CRD_00387	CRC_01215
186681299		hypothetical protein	all1340	no	no
186681300		hypothetical protein	all1339	no	no
186681350		PpiC-type peptidyl-prolyl cis-trans isomerase	alr1613	no	CRC_02567
186681409		HEAT repeat-containing PBS lyase	alr2986	CRD_00077	CRC_02169
186681476		peptidoglycan binding domain-containing protein	alr4984	CRD_02468	CRC_02058
186681631		nuclease	all2918	CRD_01392	CRC_01535
186681697	core set	hypothetical protein	alr2393	CRD_02002	CRC_01280
186681814		hypothetical protein	all3643	CRD_01982	CRC_01258
186681958	core set	hypothetical protein	all1729	CRD_02583	CRC_00038
186682138	core set	peptidase S48, HetR	alr2339	CRD_01519	CRC_03184
186682240	core set	PatU3	alr0101	CRD_02293	CRC_02800
186682241	core set	HetZ	alr0099	CRD_02292	CRC_02801
186682787		hypothetical protein	alr1555	no	no
186682808		peptidoglycan binding domain-containing protein	all1861	no	no
186683172		hypothetical protein	all5122	CRD_01021	CRC_02539
186683174		hypothetical protein	all2077	no	no
186683213		hypothetical protein	all1154	CRD_00512	CRC_00964
186683474		GDSL family lipase	all0976	no	no
186683904		hypothetical protein	all0215	CRD_00210	CRC_03261
186683953		GDSL family lipase	all1288	no	no
186684054		hypothetical protein	asr1049	no	no
186684093	core set	hypothetical protein	all2344	CRD_00085	CRC_00676
186684579	core set	hypothetical protein	all2320	CRD_01527	CRC_01389
186684586		hypothetical protein	all5091	CRD_02655	CRC_00188
186685511	core set	hypothetical protein	alr4863	CRD_02120	CRC_01594
186685539		NUDIX hydrolase	alr2015	CRD_01916	CRC_01834
186685973		hypothetical protein	all1007	no	CRC_00879
186685974		hypothetical protein	all1006	no	CRC_00878
186686413		hypothetical protein	all4622	no	no

Insights into evolution of the 10 core genes were obtained by phylogenetic analysis (Figure S6). The trees clearly show the high phylogenetic affiliation between CS-505 and D9, supported by bootstrap values of 100%, and the closest association to the CS-505/D9 branch to *Nostoc azollae*, supported for 9 of the core genes. All the core genes support the monophyly of heterocystous cyanobacteria (belonging to subsection IV), consistent with previous reports based on 16S rRNA, *hetR* [3] or phylogenomics [4], [5]. It is remarkable that seven of the core genes have an ortholog in *Synechococcus* sp. PCC 7335. The closest relationship of this organism with filamentous cyanobacteria has been reported by 16S rRNA phylogeny [36], and an ortholog of HetR was also described [31]. Our results strongly indicate that this organism could be the closest ancestor of filamentous cyanobacteria.

Although our BLAST analysis selected the gene pair CRC_00038/CRD_02583 as part of the core genes, only the branch formed by heterocystous cyanobacteria is resolved on the phylogenetic tree. Non-heterocystous cyanobacteria cluster with unicellular taxa, suggesting that this gene is part of a different family and therefore was removed from the core.

### Nitrogen Fixation

Diazotrophy is an ancient character, marking the lineage from which filamentous cyanobacteria seem to have evolved [3], [5]. In comparing the gene repertoire of all available diazotrophic species with that of non-diazotrophic, we ended up with 49 genes that were present in at least eight of the nine genomes we chose for the first analysis. These 49 genes comprise the upper limit of true inventions at this evolutionary juncture. As the functional classification confirms, most of the gene products are indeed involved in N_2_ fixation (Table 5). The data set can be dissected into three distinctive categories: 1) the *nif* cluster and related genes, 2) the uptake hydrogenase gene cluster (*hupSL*) and endopeptidase specific for the uptake hydrogenase *hupW*, and 3) finally, a set of genes involved in general metabolism and hypothetical proteins.

**Table 5:** Genes present in N_2-_ fixing species.

Npun	Gene		Gene product	Anab	D9	CS-505	Absent in
186680715			glycerophosphoryl diester phosphodiesterase	all1051	CRD_01538	CRC_01381	SynJA3, SynJA2, Cya7425
186680864	*patB*	core set	4Fe-4S ferredoxin iron-sulfur binding domain-containing protein	all2512	no	CRC_01763	
186680869			hypothetical protein	alr2517*	no	CRC_03082	SynJA3, SynJA2, Cya8801
186680870			cupin 2 domain-containing protein	alr2518*	no	CRC_03081	SynJA3, SynJA2, Mcht
186680871			nitrogenase-associated protein	alr2520*	no	CRC_03080	Mcht
186680875			hypothetical protein	asr2523*	no	CRC_02152	SynJA3, SynJA2, Cya7425
186680876			hypothetical protein	alr2524*	no	CRC_02151	SynJA3, SynJA2, Cya7425, Mcht
186680892			NHL repeat-containing protein	alr0693	no	no	SynJA3, SynJA2, Cya7425, Mcht
186680893			Rieske (2Fe-2S) domain-containing protein	alr0692	no	no	
186680895			hypothetical protein	asr0689	no	CRC_01692	SynJA3, SynJA2, Cya7425, Mcht
186680897	*hupS*		Ni Fe-hydrogenase small subunit, HupS	all0688	no	CRC_02736	SynJA3, SynJA2, Cya7425, Mcht
186680898	*hupL*		Ni Fe-hydrogenase large subunit, HupL	all0687	no	CRC_02737	SynJA3, SynJA2
186680903	*hupW*		hydrogenase maturation protease	alr1423	no	CRC_01049	SynJA3, SynJA2, Cya7425
186680908	*feoA*		FeoA family protein	asl1429	no	CRC_02875	Tery, Lyng, Nspu
186680909	*fdxH*		ferredoxin (2Fe-2S)	all1430	no	CRC_02876	Mcht
186680910	*hesB*		iron-sulfur cluster assembly accessory protein	all1431	no	CRC_02877	SynJA3, SynJA2
186680911	*hesA*		UBA/THIF-type NAD/FAD binding protein	all1432	no	CRC_02878	SynJA3, SynJA2
186680912	*nifW*		nitrogen fixation protein	all1433	no	CRC_02879	Mcht
186680913			protein of unknown function DUF683	asl1434	no	CRC_02880	Tery
186680914			protein of unknown function DUF269	all1435	no	CRC_02881	Mcht
186680915	*nifX*		nitrogen fixation protein	all1436	no	CRC_02882	Mcht
186680916	*nifN*	core set	nitrogenase molybdenum-iron cofactor biosynthesis protein NifN	all1437	no	CRC_02883	
186680917	*nifE*	core set	nitrogenase MoFe cofactor biosynthesis protein	all1438	no	CRC_02884	
186680918			Mo-dependent nitrogenase family protein	all1439	no	no	Tery
186680919	*nifK*	core set	nitrogenase molybdenum-iron protein beta chain	all1440	no	CRC_02885	
186680550	*nifD*	core set	nitrogenase molybdenum-iron protein alpha chain	all1454	no	CRC_02886	
186680941	*nifH*	core set	nitrogenase iron protein NifH	all1455	no	CRC_02887	
186680943	*nifU*	core set	Fe-S cluster assembly protein NifU	all1456	no	CRC_02888	
186680944	*nifS*	core set	Nitrogenase metalloclusters biosynthesis protein NifS	all1457	no	CRC_02889	
186680946	*nifB*	core set	nitrogenase cofactor biosynthesis protein	all1517	no	CRC_02891	
186680953	*cysE*		serine acetyltransferase	alr1404	no	no	SynJA3, SynJA2
186680954			hypothetical protein	asr1405*	no	no	
186680955		core set	hypothetical protein	asr1406*	no	CRC_02071	
186680956	*nifV*		homocitrate synthase	alr1407	no	CRC_02070	Tery, Mcht
186680957	*nifZ*		NifZ family protein	asr1408	no	CRC_02069	Mcht
186680958	*nifT*		NifT/FixU family protein	asr1409	no	CRC_02068	Mcht
186680959			hypothetical protein	alr1410	no	CRC_02067	Tery
186682206			hypothetical protein	all0969	no	no	SynJA3, SynJA2
186682693			ribokinase-like domain-containing protein	alr4681	CRD_01205	CRC_01938	SynJA3, SynJA2, Cya7425
186683057			hypothetical protein	alr0857	no	no	SynJA3, SynJA2
186683906			pathogenesis related protein-like protein	all0217	no	CRC_03259	SynJA3, SynJA2, Cya7425, Mcht
186684105			glycosyl transferase, group 1	all1345	CRD_02459	no	SynJA3, SynJA2, Cya7424
186684241			hypothetical protein	all4434	CRD_01931	CRC_02458	SynJA3, SynJA2, Cya7425
186685158			phosphoglycerate mutase	alr2972	CRD_00352	CRC_03094	Tery, SynJA3, SynJA2, Cya7425
186685476			hypothetical protein	asl0163	no	no	SynJA3, SynJA2
186685625			hypothetical protein	all3713*	no	no	SynJA3, SynJA2, Cya7425, Cya8801
186685845			hypothetical protein	asl0597	no	no	SynJA3, SynJA2, Cya7425
186686227			Arginyl tRNA synthetase anticodon binding	all3951	CRD_01597	CRC_03274	
186686347			cytochrome P450	all1361	no	no	SynJA3, SynJA2, Cya7425

*Genes that show regulation in *Anabaena* under N_2_- depletion [38].

Three genes coding for hypothetical proteins normally located between *hupSL* and maturation hydrogenase gene clusters (*hypABCDEF*) [37] belong to the group of 49 genes, suggesting their key role in N-metabolism. Part of the set also comprises genes found to be up-regulated under N-depletion in *Anabaena* [38] (Table 5). Interestingly, the CS-505 strain does not have the full set of 49 genes. Genome comparison with this strain thus further narrows the set of gene products needed for diazotrophy down to only 38. This indicates that a streamlined genome like that of *C. raciborskii* may be able to dispense with some otherwise needed genes. Analysis of several further genomes to account for species variability allowed us to define an indispensable core gene set for all species. Unexpectedly, in some *Cyanothece* and extremophile *Synechococcus* genomes many of the previously found common genes were not present, e.g. the uptake hydrogenase and related genes and genes that show changes in expression in heterocysts are missing (Table 5). *Microcoleus chthonoplastes* has not been classified as a N_2_ fixing cyanobacterium, however, its genome contains the *nifHDK* and *nifEN* gene clusters with similarity to δ-proteobacteria rather than cyanobacteria suggesting that this cluster was transferred by HGT [39]. When we considered the *Cyanothece*, *Synechococcus* and *M. chthonoplastes* PCC 7420 genomes, our core set was highly reduced to 10 genes: the *nif* gene cluster and related genes and *patB*. PatB has an N-terminal- ferredoxin and a C-terminal helix-turn-helix domain suggesting its function as a redox-sensitive transcription factor [40]. Furthermore, in *Anabaena*, a *patB* deletion mutant was completely defective for diazotrophic growth, but in the wildtype, its expression was restricted to heterocysts [41]. The presence of *patB* as part of the core gene for diazotrophic cyanobacteria suggests that this gene is also essential in unicellular and non-heterocystous diazotrophic cyanobacteria.

Further evidence for the correct logic of our approach is provided from genomic data of the D9 strain. This cyanobacterium has lost the ability to fix N_2_, and as we show in our analysis, it lost genes involved in this process. Indeed, only 6 of the 49 genes were detected in this strain. The gene products of these are related to phosphoglycerol metabolism and therefore are possibly involved in membrane degradation/synthesis. If they were once involved in N_2_ fixation they likely now fulfill indispensable functions, such that a loss would lead to decreased fitness or lethality.

### Heterocyst Development

The process of heterocyst differentiation has been described in detail only in *Anabaena* and *Nostoc punctiforme* ATCC 29113, which develop intercalated heterocysts in a specific pattern [42]. There are no published studies on heterocyst differentiation in cyanobacterial genera that develop terminal heterocysts, such as *Cylindrospermopsis* or *Cylindrospermum*. Our comparative genomic screen for genes restricted to heterocyst-forming species delivered an overwhelming number of 149 genes (Table 3, Table S3). This high number can be explained by the fact that only a few genomes of heterocyst-forming species are currently known, but they are also rather closely related. Some of the 149 genes may be involved in the formation of intercalating heterocysts. If we include the genomes of our strains in this analysis only 58 unique genes remain as common to all heterocyst-forming species. A further slight reduction in gene numbers to 41 was achieved by including the symbiont *Nostoc azollae* in the analysis. Of these genes, only one, *patN*, is currently described as involved in pattern formation.

In *Anabaena*, 77 genes are described as having a function in heterocyst differentiation, but only 55 of these have a homolog in the *C. raciborskii* CS-505 genome (Table S4). Surprisingly, one of the genes thus far seen as essential for heterocyst differentiation, *hetC*, is absent. This gene represses the expression of *ftsZ* in early stages of heterocyst differentiation in *Anabaena*, and the Δ*hetC* mutant generates multiple clusters of uncompromised pro-heterocysts along the filament that are capable of cell division and elongation [43]. Other genes related with key steps in heterocyst differentiation that are absent in CS-505 include: *ccbP*, whose product has been shown to regulate the calcium availability for heterocyst formation and negatively regulate the heterocyst differentiation [44]; *hetL*, a positive regulator of the differentiation process that interferes with *patS* inhibition process [45]; and *hetN*, a suppressor of heterocyst differentiation involved in the maintenance of the delayed heterocyst spacing pattern [46]. Lack of these genes again points to streamlining in the *C. raciborskii* genome and could possibly be attributed to a terminal rather than an intercalating heterocyst formation.

PatS, or a pentapeptide PatS-5, have been proposed to be diffusible molecules acting as an inhibitor of heterocyst differentiation [47]. In the current model, HetR activates the expression of *patS*, and PatS or a derivative diffuses laterally to inhibit the differentiation of the neighboring cells by acting negatively on the DNA-binding activity of HetR [42]. No CDS for a protein with the typical characteristics described for PatS, i.e. a diffusible penta-peptide with distinctive last C-terminal amino-acid, was found in the CS-505 and D9 genomes. Zhang *et al*., showed that a *patS*-like CDS of the non-diazotrophic *Arthrospira platensis* could complement a *patS* mutant of *Anabaena*, despite the fact that its conserved penta-peptide (RGSGR) was not in the last amino acids of the predicted protein [31]. Thus, other penta-peptide-containing proteins could have taken over the function of PatS in *C. raciborskii*. A deeper analysis for proteins containing the penta-peptide revealed a CDS that had the penta-peptide in the C-terminal region in the genomes of CS-505 and D9. We propose therefore that if *patS* exist in these cyanobacteria, the most likely candidates are CRC_02157 and CRD_02133 in CS-505 and D9, respectively.

Our analysis also revealed that 39 of the 55 heterocyst differentiation-related genes in CS-505 are also present in the non-heterocystous *R. brookii* D9 genome (Table S4). In *Nostoc punctiforme*, the cellular differentiation pathways for hormogonia, akinetes and heterocysts are reported to have genes with common expression profiles [48]. Since we observed akinetes in strain D9 by optical microscopy, it is most likely that these genes with described functions in heterocyst differentiation have additional functions and/or are involved in other cellular differentiation processes such as akinete formation.

Terminal cell differentiation was evident from electron micrographs (Figure 1) of both strains. Nevertheless, terminal cell differentiation in D9 resembles the morphology of an immature heterocyst of CS-505 (not shown), suggesting incomplete heterocyst development. The final steps in heterocyst development involve the synthesis and deposition of an inner glycolipid layer and then covering with a polysaccharide envelope [42]. These layers isolate the newly differentiated cell from external oxygen. The inner glycolipid layer is synthesized by a cluster of genes involving a polyketide synthase (PKS) pathway and glycosyltransferases in *Anabaena* [49], [50]. *Cylindrospermopsis raciborskii* CS-505 contains most of these genes (*hglEGDCA* and *hetM*), with the exception of the aforementioned *hetN* (Figure 1, Table S4). In *Anabaena*, *hetN* is located adjacent to the phosphopanteinyltransferase *hetI*. In CS-505, however, a sequence similar to *hetI* is located at a different locus, implying structural differences in the glycolipid layer between the CS-505 and *Anabaena* heterocysts. In any case, further analysis must be done to understand the implications on the glycolipid structure of *C. raciborskii*.

The N_2_-fixation and heterocyst glycolipid clusters are not present in the D9 genome (Figure 1). Only *hetI* is present in the D9 genome, which suggests a different role of this gene in this strain. Strain CS-505 and D9 genomes do, however, contain an identical gene arrangement of the genes necessary for the synthesis of the polysaccharide envelope of the heterocysts [51] (Table S4, Figure S7). Since neither heterocyst formation nor N_2_-fixation occur in D9 it seems unlikely that the polysaccharide layer is properly deposited in the terminal D9 cells. Indeed, when we stained for polysaccharide with Alcian blue, D9 filaments were homogeneously but only slightly stained (Figure 1), indicating that polysaccharide was being synthesized, but not as a heterocyst-protective layer.

Since D9 shows features of heterocyst formation, we expect that most gene products responsible for this trait are also encoded in this strain. Indeed, only five genes of the smallest common set of 41 genes are not present in the D9 strain. The lack of these five genes could be entirely responsible for the incomplete heterocyst formation in D9. Unfortunately, no function is as yet assigned to these genes. Most of the other genes have also no assigned function for their gene products. Although all five genes are annotated as conserved hypothetical proteins, the intensive studies of N-metabolism and heterocyst development in *Anabaena* allowed us to search for possible functions of these five genes absent in D9. Indeed, we found possible functions for three genes. Both alr2522 and all0721 were shown to be up- regulated in a mutant expressing HE0277, a homolog of the sigma factor *sigJ* of alr0277 that confers resistance to desiccation by up-regulating genes involved in polysaccharide synthesis [52]. Furthermore, all1814 was up-regulated after 8 h of N-depletion and showed no significant regulation in an *nrrA* mutant, an N-regulator that facilitates heterocyst development [53]. Together this evidence suggests that alr2522 and all0721 are involved in polysaccharide biosynthesis and that all1814 is related to a stage of heterocyst development. Their absence in D9 makes them ideal targets for further functional studies on heterocyst development.

### Conclusions

The innovations of diazotrophy, filamentous growth, photosynthesis and the capacity for cellular differentiation are major defining events in the evolution of cyanobacteria. Given that the free-living cyanobacteria *C. raciborskii* CS-505 and *R. brookii* D9 have the smallest known genomes among filamentous cyanobacteria, they are ideal subjects for exploration of the development and modifications of these characteristics among cyanobacteria. In spite of their relatively small genomes, these strains are nevertheless capable of cell differentiation. We are likely observing evidence of genetic streamlining, pointing towards the minimum set of genes required for these traits. Remarkably, strain CS-505 is able to develop a functional heterocyst without supposedly “essential” genes, such as, *hetC, hetN, hetL* and *ccbp*.

The *C. raciborskii* CS-505 and *R. brookii* D9 strains have geographically disjunct origins within tropical freshwater ecosystems. We expect that they have been genetically isolated and hence have evolved independently. Nevertheless, on the basis of 16S rRNA they are virtually identical and form a monophyletic cluster, with a close phylogenetic affiliation among filamentous cyanobacteria. The morphological criteria originally used to discriminate between these strains and to assign them to different genera obviously reflect differential genetic editing primarily associated with cell differentiation and functional heterocyst formation rather than their phylogenetic relationships. With respect to this high similarity in 16S rRNA, as well as that revealed in our phylogenomic analysis (>90% identity in their 2539 shared genes), we propose that these strains are congeneric. This evidence suggests that the strain differences may represent an example of allopatric speciation.

Our genomic analysis provides support for the idea that cyanobacteria are capable of evolving according to highly diverse strategies for genomic organization and adaptive mechanisms. Whereas CS-505 shows evidence of phenotypic plasticity and has a more elaborate genome, perhaps via gene acquisition and rearrangement, D9 has apparently adapted by losing genes and avoiding horizontal gene transfer. These alternative strategies have important implications for the adaptive radiation of filamentous cyanobacteria and at least partially account for their evolutionary success in a multitude of environments over enormously long time-scales.

## Materials and Methods

### Isolation and Culture of Cyanobacterial Strains

*Cylindrospermopsis raciborskii* strain CS-505 was clonally isolated in 1996 from a water supply at the Solomon Dam, Australia [54] and obtained from the culture collection of the Commonwealth Scientific and Industrial Research Organization (CSIRO), Australia. *Raphidiopsis brookii* D9 (originally classified as *C. raciborskii*) was isolated from a mixed plankton sample collected in 1996 from the Billings freshwater reservoir near Sao Paulo, Brazil and subsequently recloned from a single filament. Strain CS-505 produces cylindrospermopsin (CYN) and deoxy-cylindrospermopsin (doCYN) [22], [54], but no PSP toxins. Strain D9 constitutively produces the following PSP toxins, as confirmed by LC-MS/MS: saxitoxin (STX), C-11 O-sulfated gonyautoxins (GTX2/3), and their respective decarbamoyl derivatives (dcSTX and dcGTX2/3) as minor components [22], [55].

The non-axenic cultures were grown in 250 ml flasks containing 100 ml of MLA growth medium [55] without aeration at 23°C under fluorescent light at a photon flux density of 35 µmol m^−2^ s^−1^ on a 12:12 h light/dark photocycle. To minimize bacterial contamination several wash steps were performed after harvesting and the absence of eubacterial DNA was checked by PCR as previously described [22].

### Preparation and Sequencing of Genomic DNA

Long strands of genomic DNA were obtained by purifying DNA embedded on low melting point (LMP) agarose plugs. Intact chromosomal DNA embedded on agarose plugs was obtained from 100 ml of healthy cultures in mid-exponential growth phase as previously described [22].

Sequencing was conducted with the BigDye kit from ABI (Foster City, USA) using standard forward and reverse primers; pre-assembly trimming was performed with a modified version of Phred [56], [57].

Genomic libraries for the 454/gs20 system were prepared according to the manufacturer's protocols (454 Life Sciences Corporation, Branford, CT, USA). Three runs each were performed on the 454/gs20 sequencing system. All 454/gs20 sequence data were assembled according to species-specific criteria with the newbler assembler software (http://www.454.com). The Sanger-based sequencing reads were assembled onto this backbone. Clone gaps then were filled by a primer walking strategy with custom primers. The genome sequences of CS-505 and D9 were deposited in the NCBI genome database under the main accession numbers: ACYA00000000 (CS-505) and ACYB00000000 (D9).

### Repeat Analysis

For the CS-505 genome we calculated all supermaximal repeats. A supermaximal repeat is defined as follows:

A pair of substrings R = ((i_1_, j_1_), (i_2_, j_2_)) is a *repeated pair* if and only if (i_1_, j_1_)≠(i_2_, j_2_) and S[i_1_..j_1_] = S[i_2_..j_2_]. The length of R is j_1_ - i_1_+1. A repeated pair ((i_1_, j_1_), (i_2_, j_2_)) is called *left maximal* if S[i_1_ - 1]≠S[i_2_ - 1] and *right maximal* if S[j_1_+1]≠S[j_2_+1]. A repeated pair is called *maximal* if it is left and right maximal. A substring ⌉ of S is a (*maximal*) *repeat* if there is a (maximal) repeated pair ((i_1_, j_1_), (i_2_, j_2_)) such that ⌉ = S[i_1_..j_1_]. A *supermaximal repeat* is a maximal repeat that never occurs as a substring of any other maximal repeat.

For the given contigs of *C. raciborskii* CS-505 we found 258,229 different supermaximal repeats covering 98.52% of the whole sequence.

In a second step we clustered all supermaximal repeats close to each other and with similar distances between their positions in the genome. This helps to find larger degenerated repeats because they contain several exact super maximal repeats.

For the clustering, each supermaximal repeat containing more than two copies was decomposed into all possible copy pairs. Those pairs were then clustered according to similar first positions of the first copy respectively and according to similar distances between the copies. We selected 500 nt as the maximal allowed difference between the two first positions. The maximal allowed difference between the distances was 100 nt.

With the given parameter setting we got 5,390 different clusters. For the 20 clusters with the best score ( =  total copy length * copy pair amount), we performed a motif search with at least 60% sequence identity on both strands and in both directions. All hits of the 20 clusters cover 3.94% of the whole sequence.

### Bioinformatics Analysis

The cyanobacterial taxa used for comparative genomic analyses are listed in Table 6. Using *Nostoc punctiforme* as member of the group with all traits analyzed as “template”, we performed blastp analyses against all other genomes. We applied a score threshold of 150 to get rid of spurious hits. Remaining hits were analyzed with respect to their occurrence in four different groups: non-N_2_-fixing, N_2_-fixing, filamentous N_2_-fixing and filamentous heterocyst-forming N_2_-fixing.

**Table 6:** Characteristics of the cyanobacterial taxa used for comparative genomic analyses.

Species	Morphology	Diazotrophy	Accession number	Genome sequence status
*Nostoc punctiforme* PCC 73102 (Npun)	Filamentous, heterocystous	N_2_-fixing	NC_010628	finished
*Nodularia spumigena* CCY9414 (Nspu)	Filamentous, heterocystous	N_2_-fixing	NZ_AAVW00000000	unfinished
*Anabaena* sp. PCC 7120 (Anab)	Filamentous, heterocystous	N_2_-fixing	NC_003272	finished
*Anabaena variabilis* ATCC 29413 (Avar)	Filamentous, heterocystous	N_2_-fixing	NC_007413	finished
*Nostoc azollae* strain 0708* (Nazo)	Filamentous, heterocystous	N_2_-fixing	NZ_ACIR00000000	unfinished
*Trichodesmium erythraeum* IMS101 (Tery)	Filamentous	N_2_-fixing	NC_008312	finished
*Lyngbya* sp. PCC 8106 (Lyng)	Filamentous	N_2_-fixing	NZ_AAVU00000000	unfinished
*Microcoleus chthonoplastes* PCC 7420* (Mcth)	Filamentous	N_2_-fixing	NZ_ABRS00000000	unfinished
*Arthrospira maxima* CS-328* (Amax)	Filamentous	non-N_2_-fixing	NZ_ABYK00000000	unfinished
*Crocosphaera watsonii* WH8501 (Cwat)	Unicellular	N_2_-fixing	NZ_AADV00000000	unfinished
*Cyanothece* sp. ATCC 51142 (Cya51142)	Unicellular	N_2_-fixing	NC_010546	finished
*Cyanothece* sp. PCC 8801* (Cya8801)	Unicellular	N_2_-fixing	NC_011726	finished
*Cyanothece* sp. PCC 7424* (Cya7424)	Unicellular	N_2_-fixing	NC_011729	finished
*Cyanothece* sp. PCC 7425* (Cya7425)	Unicellular	N_2_-fixing	NC_011884	finished
*Synechococcus* sp. JA-3-3Ab* (SynJA3)	Unicellular	N_2_-fixing	NC_007775	finished
*Synechococcus* sp. JA-2-3B'a(2-13)* (SynJA2)	Unicellular	N_2_-fixing	NC_007776	finished
*Synechococcus* sp. CC9311	Unicellular	non-N_2_-fixing	NC_008319	finished
*Synechocystis* sp. PCC 6803	Unicellular	non-N_2_-fixing	NC_000911	finished
*Acaryochloris marina* MBIC11017	Unicellular	non-N_2_-fixing	NC_009925	finished
*Gloeobacter violaceus* PCC 7421	Unicellular	non-N_2_-fixing	NC_005125	finished
*Microcystis aeruginosa* NIES-843	Unicellular	non-N_2_-fixing	NC_010296	finished
*Prochlorococcus marinus* MIT 9301	Unicellular	non-N_2_-fixing	NC_009091	finished
*Synechococcus elongatus* PCC 7942	Unicellular	non-N_2_-fixing	NC_007604	finished
*Thermosynechococcus elongatus* BP-1	Unicellular	non-N_2_-fixing	NC_004113	finished

*Species included in the second part of the analysis.

A further analysis was then performed with these sets including genomes from a wider range of species to get the true core sets for the traits: *Nostoc azollae* strain 0708 is a symbiotic cyanobacterium with duckweed; *Microcoleus chtonoplastes* PCC 7420 possesses multiple filaments in one mucous sheath, and *Arthrospira maxima* CS-328 belongs to Section III of the cyanobacteria. Different *Cyanothece* and *Synechococcus* strains were used to account for species variability.

## Supporting Information

Figure S1 Size estimation of D9 and CS-505 genomes by PFGE restriction analysis. Restriction profiles were obtained by Mlu I digestion. SC: Chromosomic DNA from Saccharomyces cerevisiae. Vpkx: Genomic DNA from Vibrio parahaemolyticus RIMD 2210633 digested with Not I. PFGE electrophoresis conditions are described in Stucken et al., [22].
(0.40 MB TIF)

Figure S2 Possible extrachromosomal element in the CS-505 genome. PFGE of chromosomic DNA from strains D9 and CS-505, the possible plasmid is indicated by the arrow. SC: Chromosomic DNA from Saccharomyces cerevisiae.
(0.25 MB TIF)

Figure S3 Distribution of the total unique CDS of CS-505 and D9 into Cluster of Orthologous Groups (COGs). Unique CDS were obtained by a Best-Bidirectional Hits (BBHs) search between both genomes using a 30% cutoff.
(0.57 MB TIF)

Figure S4 Repeated sequences in a repeat unit as revealed by an analysis using miropeats. The analysis was performed according to Parsons, (1995), with a threshold score of 100 [58].
(0.72 MB TIF)

Figure S5 Structure and comparison of the toxin gene clusters in CS-505 and D9 with those previously described. A. Comparison of the CYN gene cluster of strain CS-505 with the cyr gene cluster described in C. raciborskii AWT205 [12]; B. Comparison of the STX gene cluster of strain D9 with the sxt gene cluster described in C. raciborskii T3 [13]. Identical ORFs between D9/T3 and CS-505/AWT205 are depicted in white; genes involved in the biosynthesis of STX are highlighted with horizontal gray lines and shading. The ORFs unique to D9 and CS-505, with respect to T3 and AWT205, are indicated in black. Unique ORFs in T3 are represented by black horizontal stripes. ORFs outside the clusters are represented by marginal dashed lines and gray fill.
(0.10 MB PDF)

Figure S6 Phylogenetic relationships of the 10 CDS found as core in 9 filamentous cyanobacteria. Affiliations to the cyanobacterial subsections are shown in brackets. The trees were constructed with clustalX, using the Neighbor-Joining algorithm with bootstrap of 1000, only bootstrap values higher than 60% are shown over the nodes. When available, unicellular strains were used as outgroup taxa. Trees are organized according to the appearance of each CDS pair in Table 4. GenBank accession numbers are indicated after species designation (names in bold-face correspond to sequences belonging to CS-505 and D9). Species name abbreviations were used as in materials and methods with the exception of the new sequences used in phylogenetic analyses: Anab WH: Anabaena sp. WH School st. isolate; Cylin A1345: Cylindrospermum sp. A1345; Clich UTEX2014: Cylindrospermum licheniforme UTEX 2014: Nost PCC9229: Nostoc sp. PCC 9229; Anab SI: Anabaena sp. South India 2006; Nost PCC7906: Nostoc sp. PCC 7906; Nodu KAC17: Nodularia sp. KAC 17; Shof PCC7110: Scytonema hofmanni PCC 7110; Toly CCMP1185: Tolypothrix sp. CCMP1185; Cdes PCC7102: Calothrix desertica PCC 7102; Cfri PCC6912: Chlorogloeopsis fritschii PCC 6912; Chlo PCC9212: Chlorogloeopsis sp. PCC 9212; Fmus UTEX1829: Fischerella muscicola UTEX 1829; Fmus SAG 1427: Fischerella muscicola SAG 1427-1; Fmus PCC7414: Fischerella muscicola PCC 7414; Fther PCC7521: Fischerella thermalis PCC 7521; LeptoPCC73110: Leptolyngbya sp. PCC 73110; Aplat HZ01: Arthrospira platensis HZ01; Mae843: Microcystis aeruginosa NIES-843; Mae7806: Microcystis aeruginosa PCC 7806; Cya7822: Cyanothece sp. PCC 7822; Syn7002: Synechococcus sp. PCC 7002; Syn7335: Synechococcus sp. PCC 7335.
(0.39 MB PDF)

Figure S7 Comparison of the gene clusters for heterocyst polysaccharide biosynthesis. The comparison was based in the gene cluster described for Anabaena sp. PCC 7120 [51].
(0.10 MB PDF)

Table S1 List of the unique CDS of CS-505 and D9 and their classification into the different COG categories.
(0.17 MB XLS)

Table S2 Cell division genes in cyanobacteria
(0.02 MB XLS)

Table S3 Genes common to all heterocystous cyanobacteria
(0.06 MB XLS)

Table S4 83 Previously described regulatory genes present in the genomes of the terminal heterocystous cyanobacteria C. raciborskii CS-505 and the non-heterocystous R. brookii D9.
(0.06 MB PDF)

Methods S1 Supplementary Material and Methods
(0.06 MB DOC)
