# Supplementary figures and images for: The Smallest Known Genomes of Multicellular and Toxic Cyanobacteria: Comparison, Minimal Gene Sets for Linked Traits and the Evolutionary Implications
Source: PLoS One. 2010 Feb 16;5(2):e9235. doi: 10.1371/journal.pone.0009235 (PMC2821919; doi:10.1371/journal.pone.0009235)

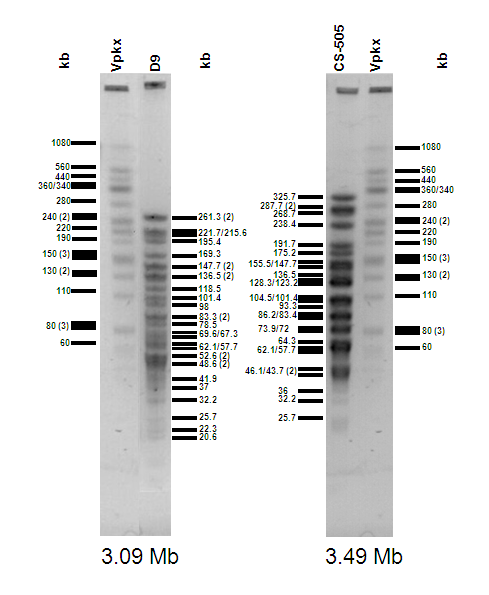

Supplement: Figure S1 — Size estimation of D9 and CS-505 genomes by PFGE restriction analysis. Restriction profiles were obtained by Mlu I digestion. SC: Chromosomic DNA from Saccharomyces cerevisiae. Vpkx: Genomic DNA from Vibrio parahaemolyticus RIMD 2210633 digested with Not I. PFGE electrophoresis conditions are described in Stucken et al., [22]. (0.40 MB TIF) [file pone.0009235.s001.tif]

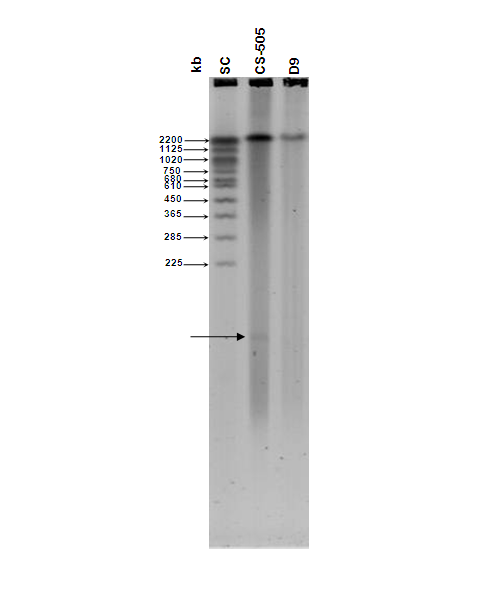

Supplement: Figure S2 — Possible extrachromosomal element in the CS-505 genome. PFGE of chromosomic DNA from strains D9 and CS-505, the possible plasmid is indicated by the arrow. SC: Chromosomic DNA from Saccharomyces cerevisiae. (0.25 MB TIF) [file pone.0009235.s002.tif]

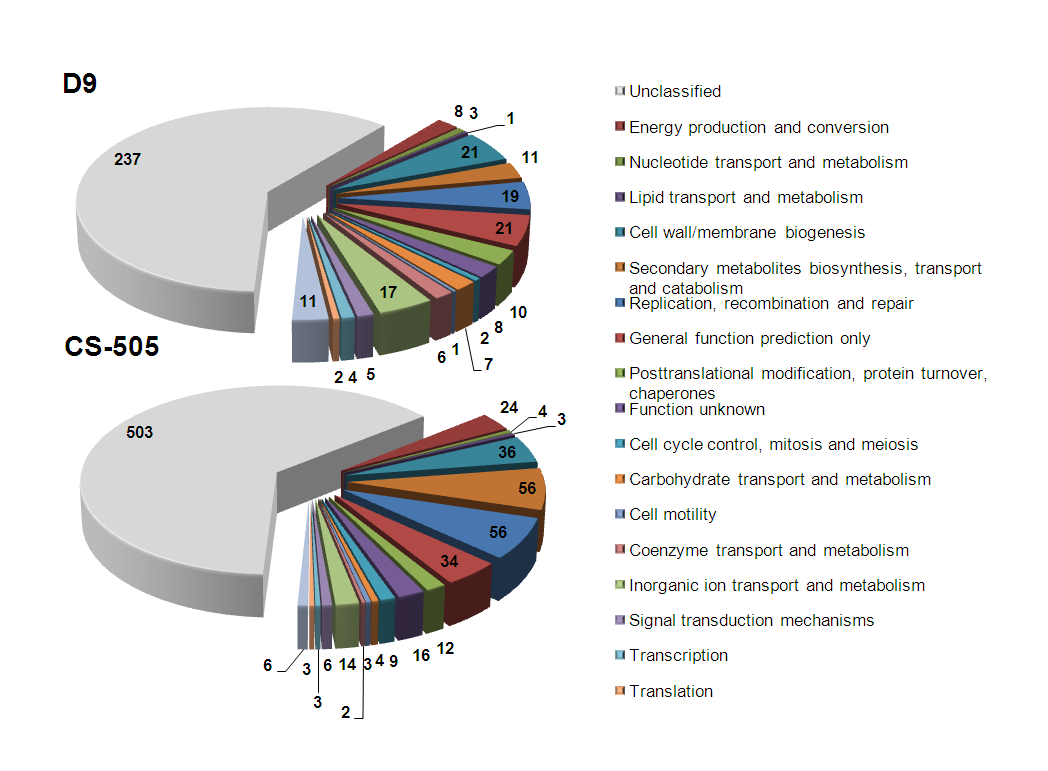

Supplement: Figure S3 — Distribution of the total unique CDS of CS-505 and D9 into Cluster of Orthologous Groups (COGs). Unique CDS were obtained by a Best-Bidirectional Hits (BBHs) search between both genomes using a 30% cutoff. (0.57 MB TIF) [file pone.0009235.s003.tif]

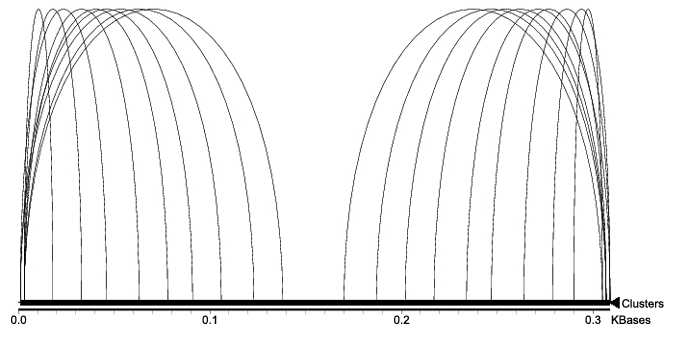

Supplement: Figure S4 — Repeated sequences in a repeat unit as revealed by an analysis using miropeats. The analysis was performed according to Parsons, (1995), with a threshold score of 100 [58]. (0.72 MB TIF) [file pone.0009235.s004.tif]

**A*****C. raciborskii* CS-505**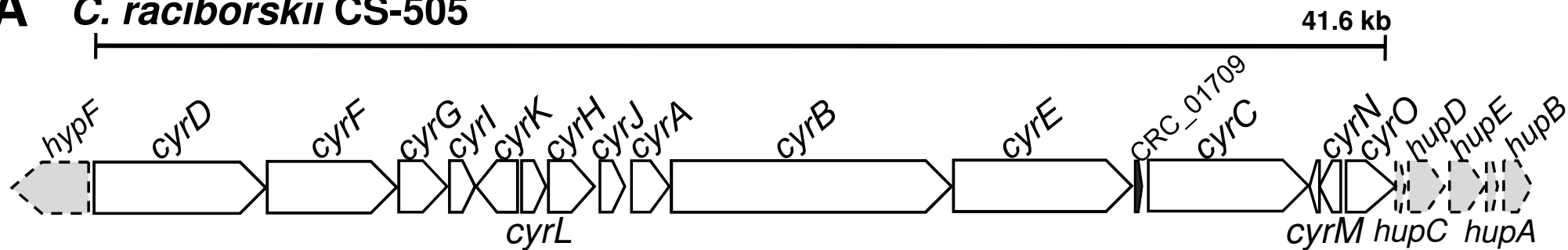***C. raciborskii* AWT205**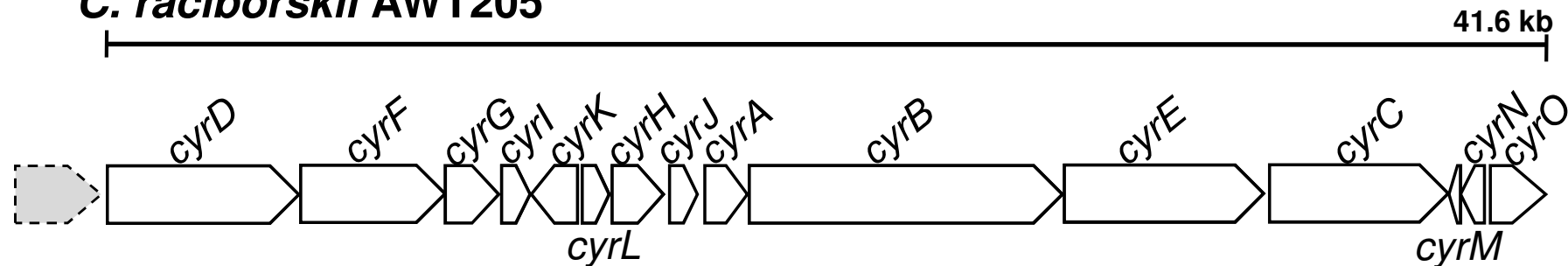**B*****R. brookii* D9**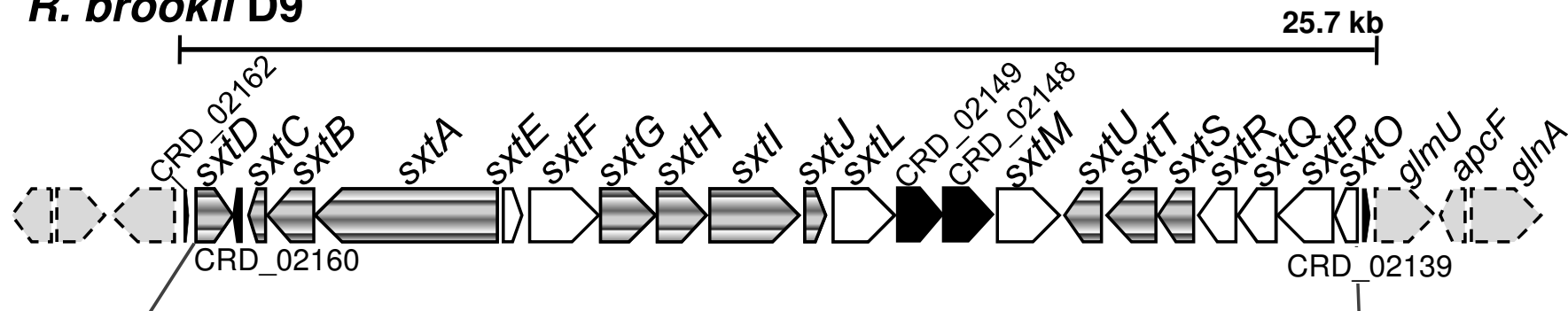***C. raciborskii* T3**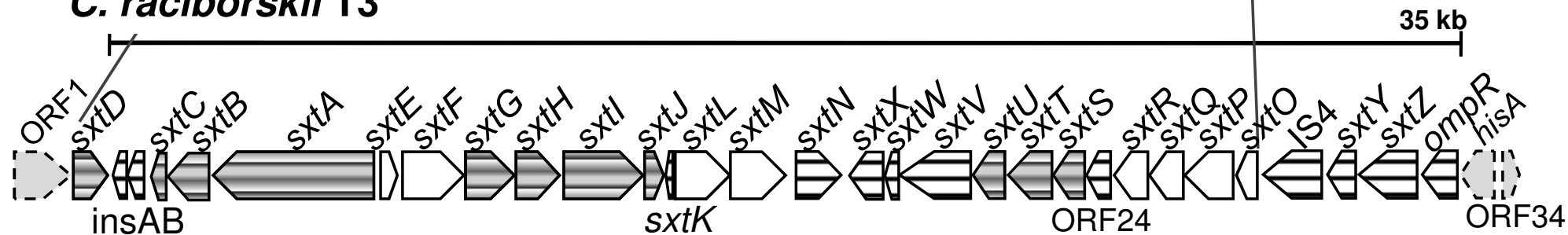

Supplement: Figure S5 — Structure and comparison of the toxin gene clusters in CS-505 and D9 with those previously described. A. Comparison of the CYN gene cluster of strain CS-505 with the cyr gene cluster described in C. raciborskii AWT205 [12]; B. Comparison of the STX gene cluster of strain D9 with the sxt gene cluster described in C. raciborskii T3 [13]. Identical ORFs between D9/T3 and CS-505/AWT205 are depicted in white; genes involved in the biosynthesis of STX are highlighted with horizontal gray lines and shading. The ORFs unique to D9 and CS-505, with respect to T3 and AWT205, are indicated in black. Unique ORFs in T3 are represented by black horizontal stripes. ORFs outside the clusters are represented by marginal dashed lines and gray fill. (0.10 MB PDF) [file pone.0009235.s005.pdf]

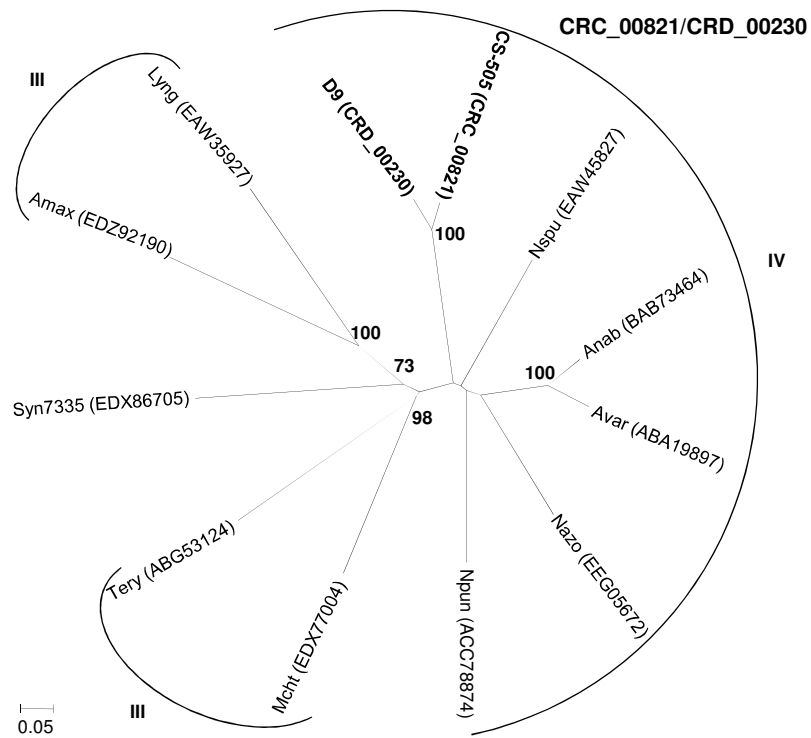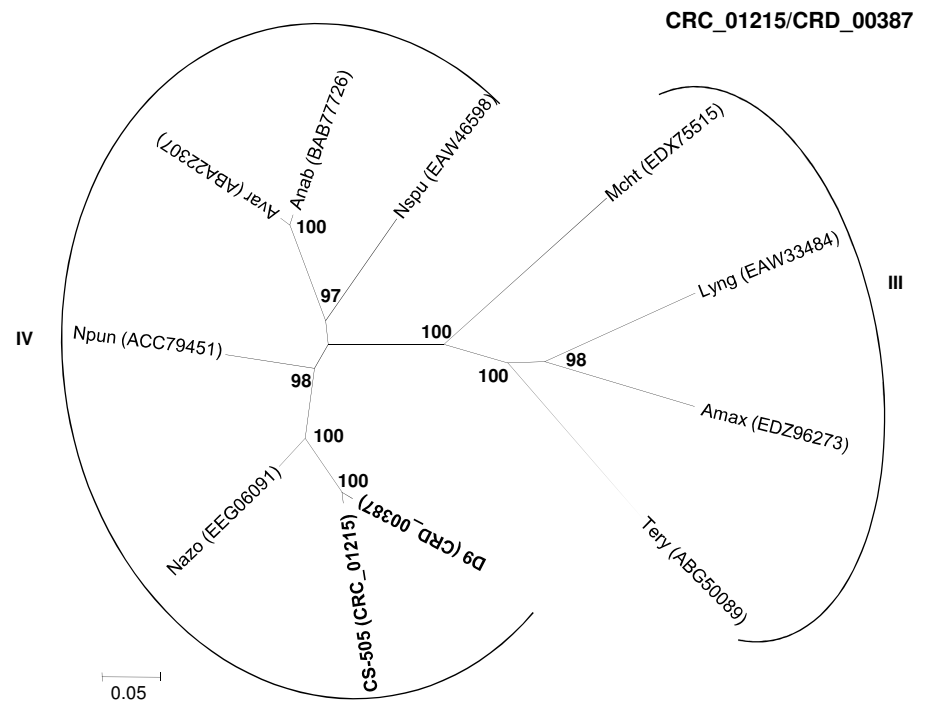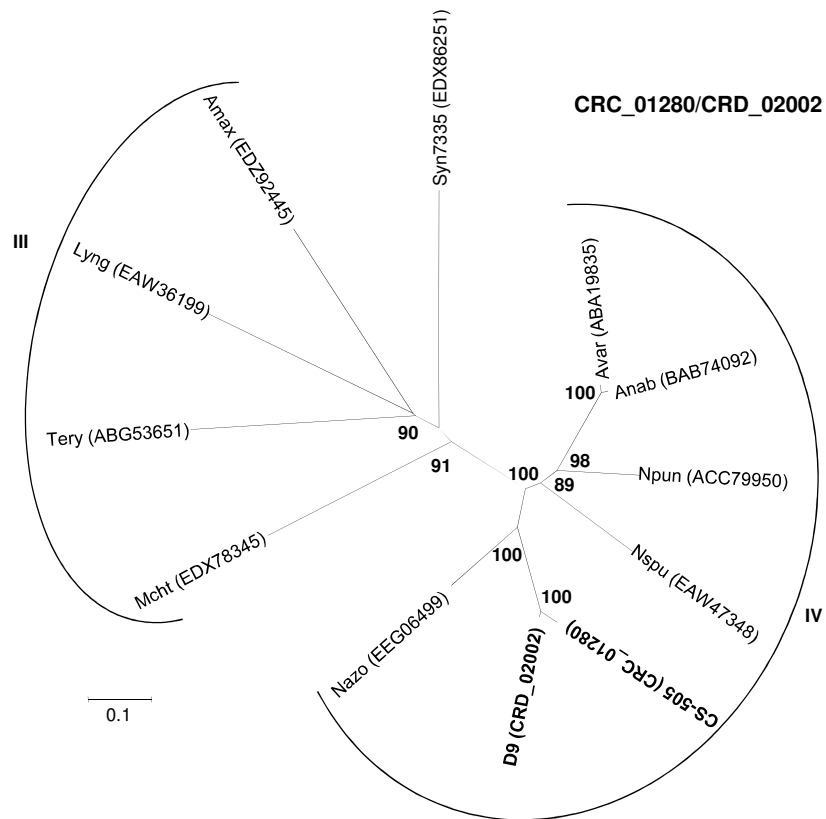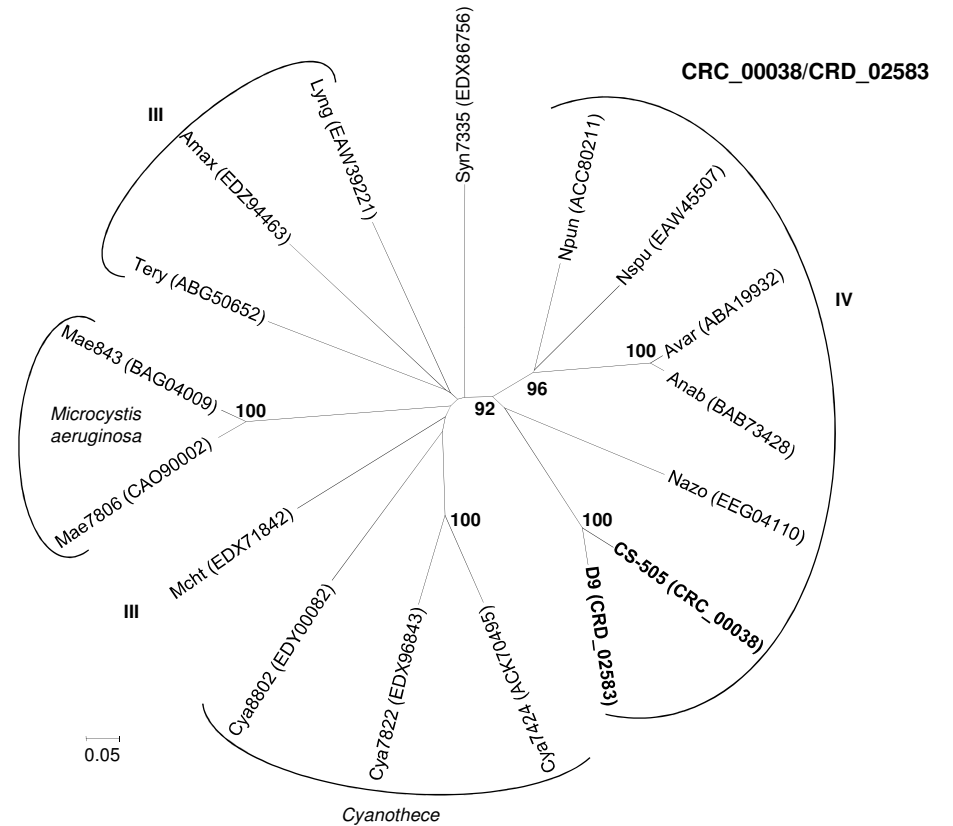

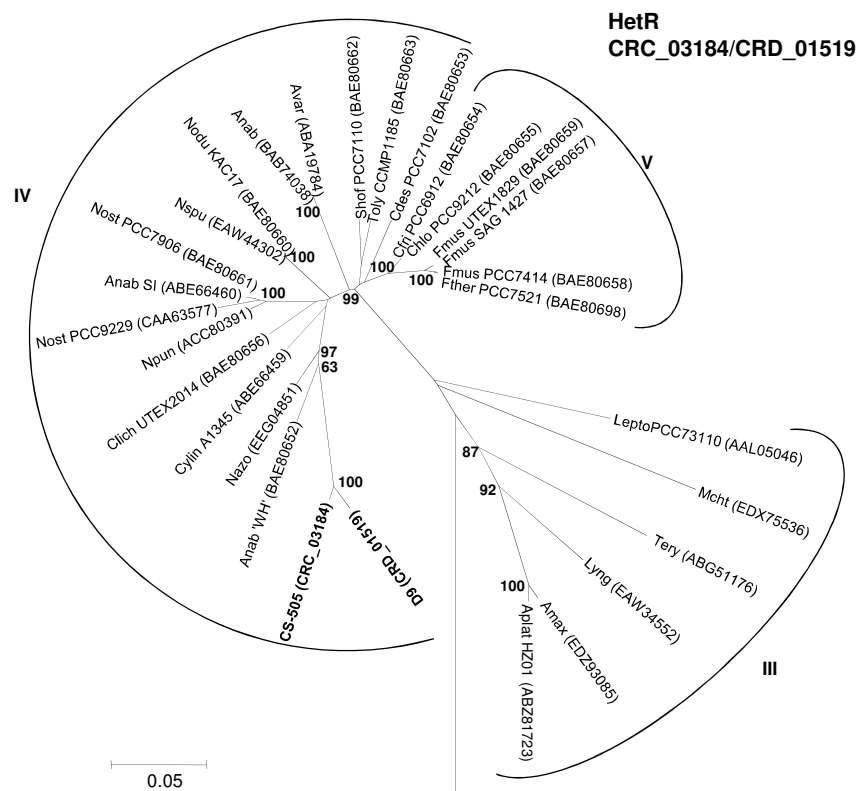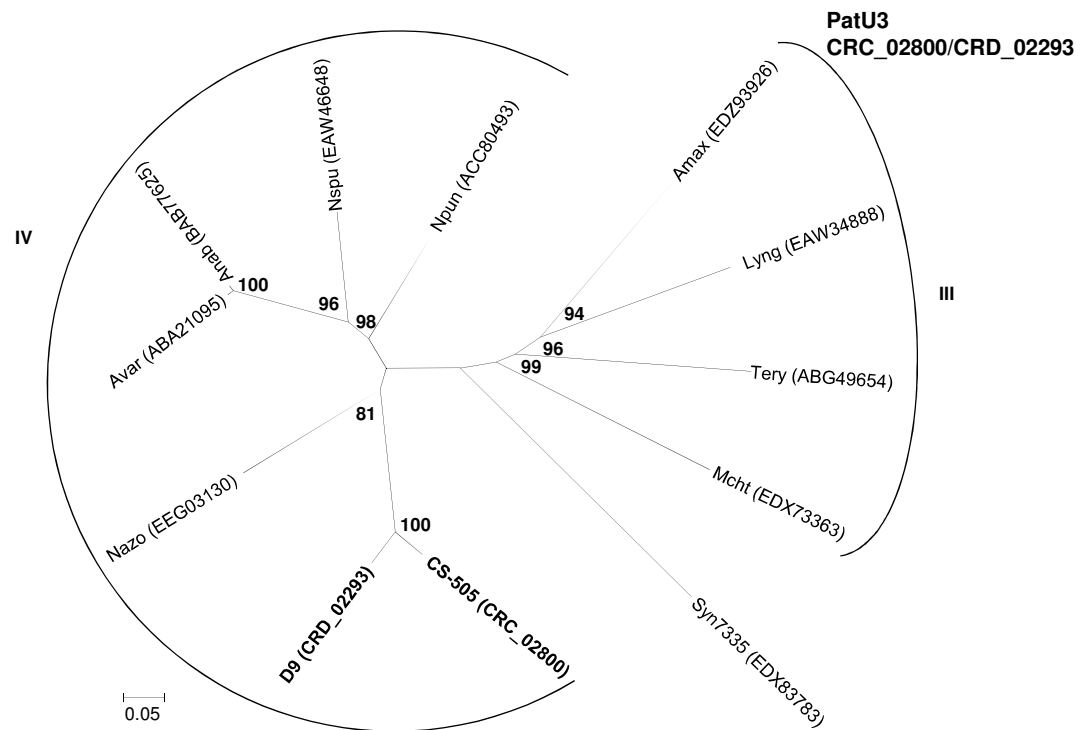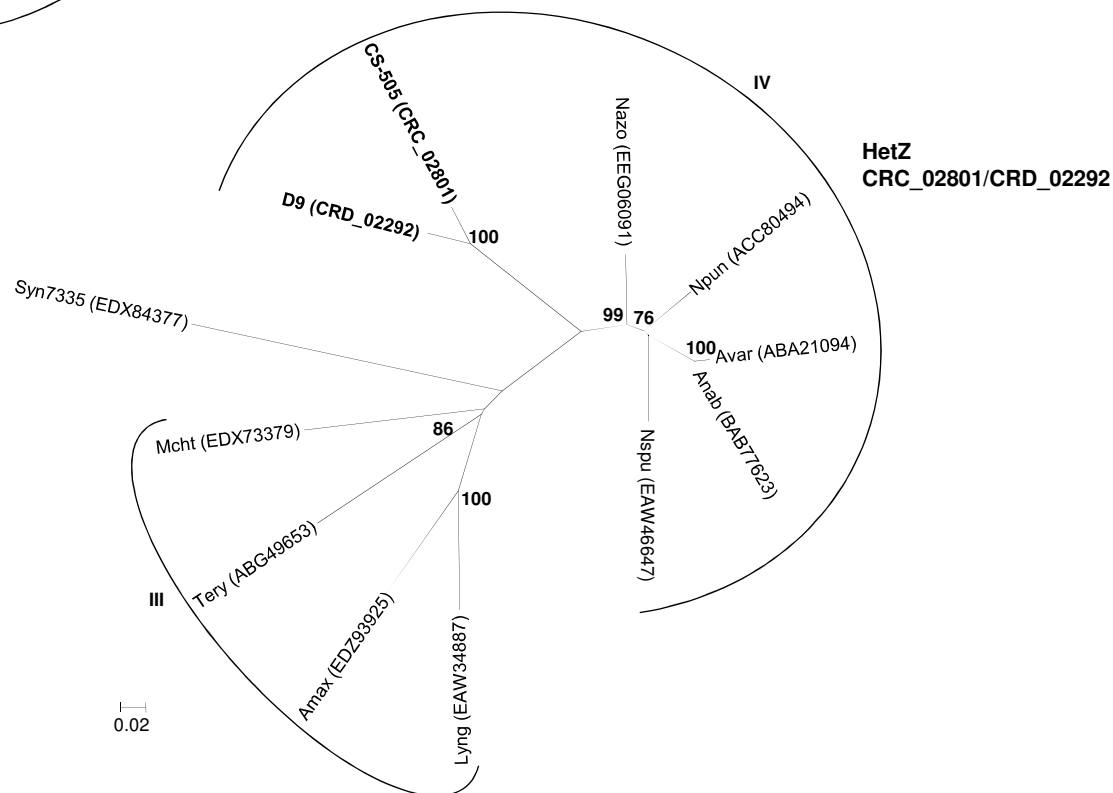

CRC\_00676/CRD\_00085

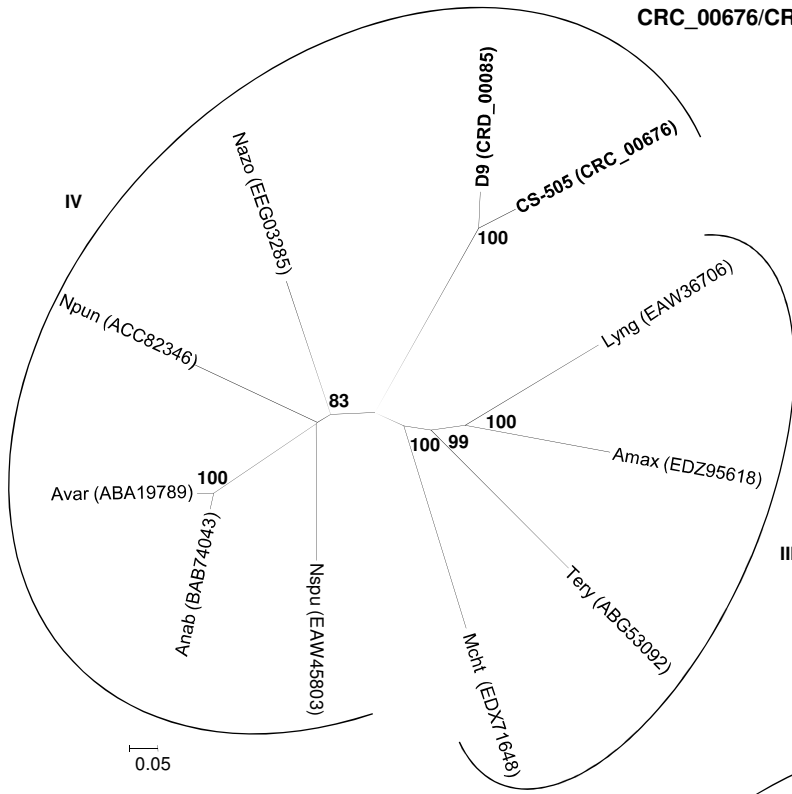

CRC\_01389/CRD\_01527

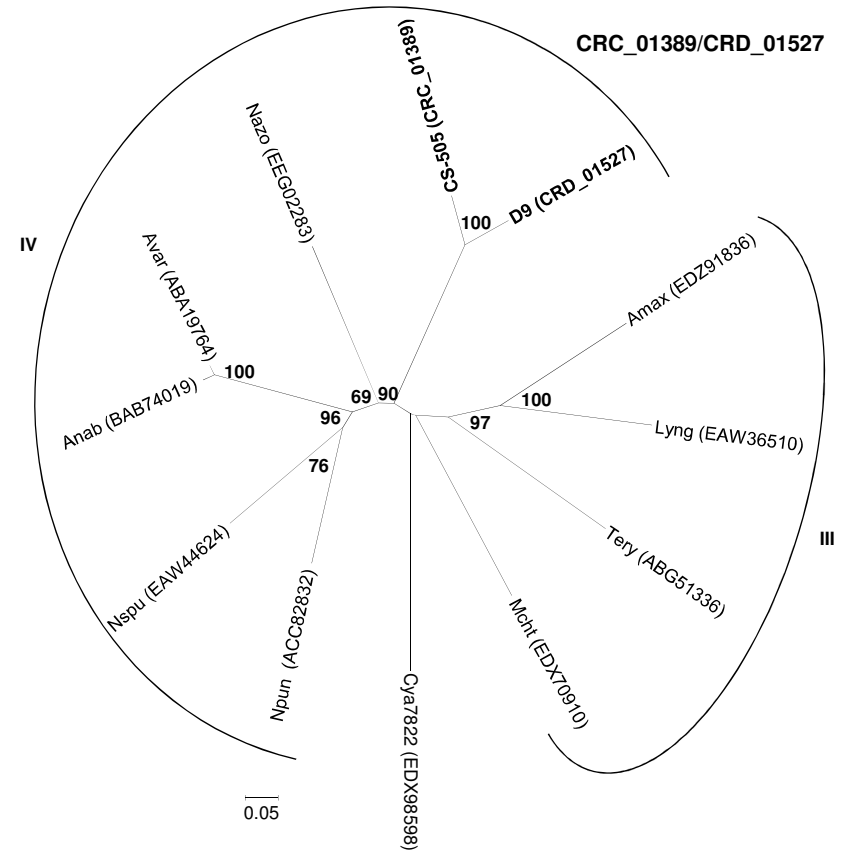

CRC\_01594/CRD\_02120

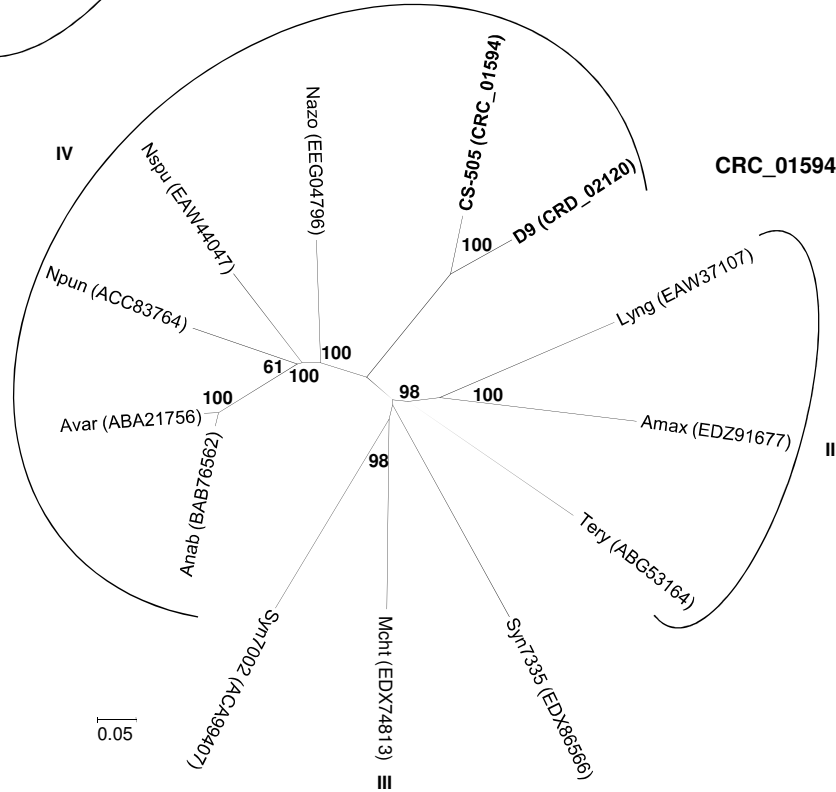

Supplement: Figure S6 — Phylogenetic relationships of the 10 CDS found as core in 9 filamentous cyanobacteria. Affiliations to the cyanobacterial subsections are shown in brackets. The trees were constructed with clustalX, using the Neighbor-Joining algorithm with bootstrap of 1000, only bootstrap values higher than 60% are shown over the nodes. When available, unicellular strains were used as outgroup taxa. Trees are organized according to the appearance of each CDS pair in Table 4. GenBank accession numbers are indicated after species designation (names in bold-face correspond to sequences belonging to CS-505 and D9). Species name abbreviations were used as in materials and methods with the exception of the new sequences used in phylogenetic analyses: Anab WH: Anabaena sp. WH School st. isolate; Cylin A1345: Cylindrospermum sp. A1345; Clich UTEX2014: Cylindrospermum licheniforme UTEX 2014: Nost PCC9229: Nostoc sp. PCC 9229; Anab SI: Anabaena sp. South India 2006; Nost PCC7906: Nostoc sp. PCC 7906; Nodu KAC17: Nodularia sp. KAC 17; Shof PCC7110: Scytonema hofmanni PCC 7110; Toly CCMP1185: Tolypothrix sp. CCMP1185; Cdes PCC7102: Calothrix desertica PCC 7102; Cfri PCC6912: Chlorogloeopsis fritschii PCC 6912; Chlo PCC9212: Chlorogloeopsis sp. PCC 9212; Fmus UTEX1829: Fischerella muscicola UTEX 1829; Fmus SAG 1427: Fischerella muscicola SAG 1427-1; Fmus PCC7414: Fischerella muscicola PCC 7414; Fther PCC7521: Fischerella thermalis PCC 7521; LeptoPCC73110: Leptolyngbya sp. PCC 73110; Aplat HZ01: Arthrospira platensis HZ01; Mae843: Microcystis aeruginosa NIES-843; Mae7806: Microcystis aeruginosa PCC 7806; Cya7822: Cyanothece sp. PCC 7822; Syn7002: Synechococcus sp. PCC 7002; Syn7335: Synechococcus sp. PCC 7335. (0.39 MB PDF) [file pone.0009235.s006.pdf]
