# Supplementary material for: The Smallest Known Genomes of Multicellular and Toxic Cyanobacteria: Comparison, Minimal Gene Sets for Linked Traits and the Evolutionary Implications
Source: PLoS One. 2010 Feb 16;5(2):e9235. doi: 10.1371/journal.pone.0009235 (PMC2821919; doi:10.1371/journal.pone.0009235)
Supplement: Figure S7 — Comparison of the gene clusters for heterocyst polysaccharide biosynthesis. The comparison was based in the gene cluster described for Anabaena sp. PCC 7120 [51]. (0.10 MB PDF) [file pone.0009235.s007.pdf]

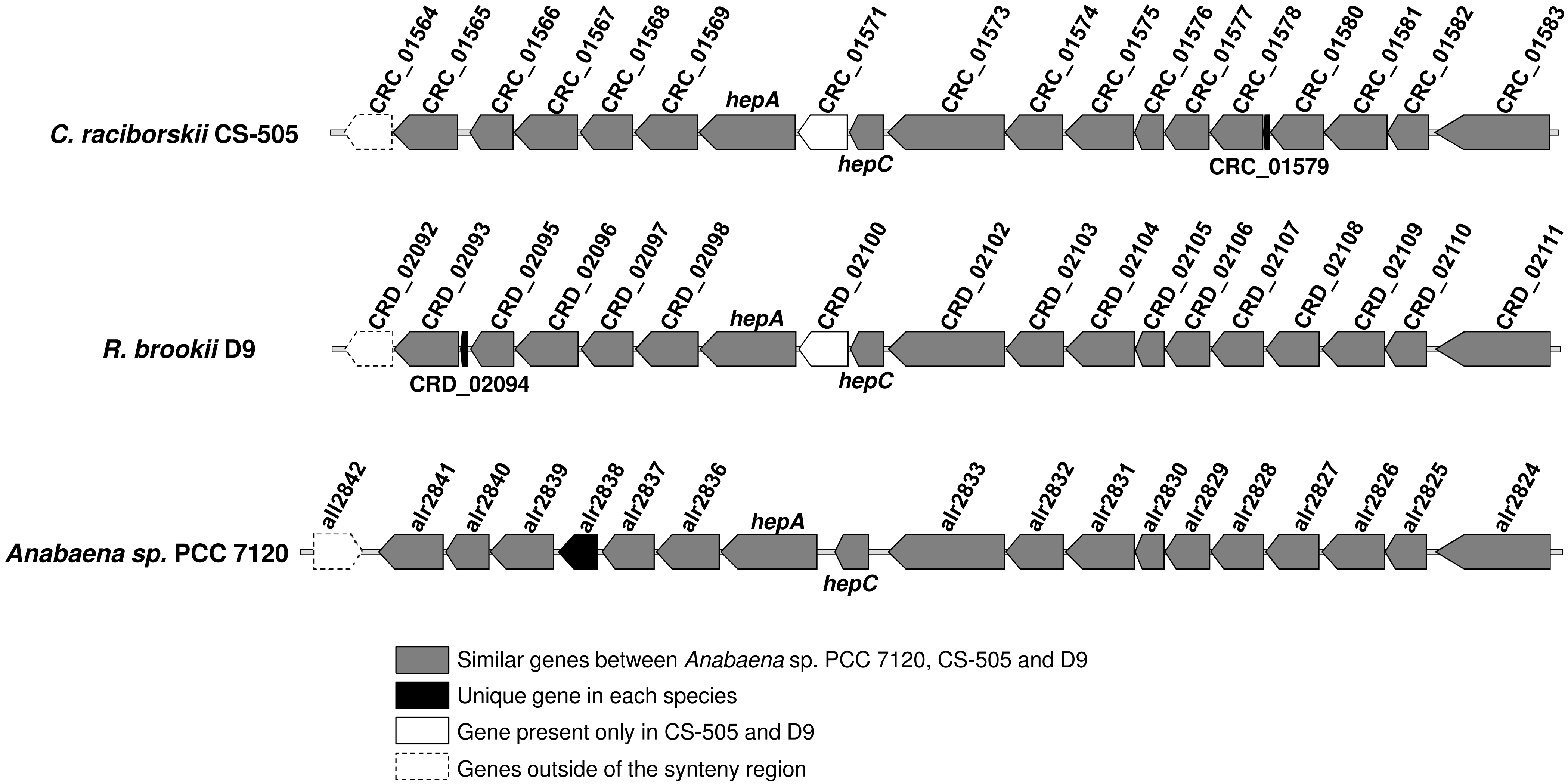

C. raciborskii CS-505
CRC_01564
CRC_01565
CRC_01566
CRC_01567
CRC_01568
CRC_01569
hepA
CRC_01571
hepC
CRC_01573
CRC_01574
CRC_01575
CRC_01576
CRC_01577
CRC_01578
CRC_01579
CRC_01580
CRC_01581
CRC_01582
CRC_01583
R. brookii D9
CRD_02092
CRD_02093
CRD_02094
CRD_02095
CRD_02096
CRD_02097
CRD_02098
hepA
CRD_02100
hepC
CRD_02102
CRD_02103
CRD_02104
CRD_02105
CRD_02106
CRD_02107
CRD_02108
CRD_02109
CRD_02110
CRD_02111
Anabaena sp. PCC 7120
all2842
alr2841
alr2840
alr2839
alr2838
alr2837
alr2836
hepA
hepC
alr2833
alr2832
alr2831
alr2830
alr2829
alr2828
alr2827
alr2826
alr2825
alr2824
Similar genes between Anabaena sp. PCC 7120, CS-505 and D9
Unique gene in each species
Gene present only in CS-505 and D9
Genes outside of the synteny region
