# Supplementary material for: The Smallest Known Genomes of Multicellular and Toxic Cyanobacteria: Comparison, Minimal Gene Sets for Linked Traits and the Evolutionary Implications
Source: PLoS One. 2010 Feb 16;5(2):e9235. doi: 10.1371/journal.pone.0009235 (PMC2821919; doi:10.1371/journal.pone.0009235)
Supplement: Table S4 — 83 Previously described regulatory genes present in the genomes of the terminal heterocystous cyanobacteria C. raciborskii CS-505 and the non-heterocystous R. brookii D9. (0.06 MB PDF) [file pone.0009235.s011.pdf]

Table S4: 83 Previously described regulatory genes present in the genomes of the terminal heterocyst forming cyanobacteria *C. raciborskii* CS-505 and the non-heterocystous *R. brookii* D9.

***Anabaena* PCC 7120**	**Gene**	***C. raciborskii* CS-505**	**Identity (%)**	***R. brookii* D9**	**Identity (%)**	**Described function/role**
all1940	*abp1*	CRC_02963	76.30	CRD_02427	77.77	Heterocyst glycolipid-layer formation [1]
all1939	*abp2*	CRC_02964	72.69	CRD_02426	72.89	
alr3608	*abp3*	CRC_00830	56.18	-	-	
alr4240	*abp4 (hlyD)*	CRC_02581	65.90	CRD_01244	64.24	
alr1010	*ccbP*	-	-	-	-	Heterocyst differentiation [2]
all0601	*cnaT*	CRC_01748	65.60	CRD_01497	64.45	*Nir* operon transcriptional regulator [3]
all0187	*conR*	CRC_00948	62.62	CRD_02925	62.46	Heterocyst functionality [4]
alr3712	*devA*	CRC_00436	80.70	-	-	Heterocyst polysaccharide formation [5-8]
alr3710	*devB*	CRC_00438	65.32	-	-	
alr3711	*devC*	CRC_00437	77.74	-	-	
alr3952	*devH*	CRC_03275	98.32	CRD_01596	98.32	
alr0442	*devRa*	CRC_02562	83.72	CRD_01268	79.84	
all1430	*fdxH*†	CRC_02876	70.40	-	-	Heterocyst ferredoxin [9]
alr3858	*ftsZ*	CRC_03281	86.34	CRD_01589	86.41	Septum formation during cell division [10]
all1691	*furA*	CRC_01433	88.07	CRD_00658	88.74	Iron metabolism regulation [11]
all2319	*glnB*	CRC_02206	92.85	CRD_00313	93.75	N_2 metabolism regulation [12]
asr3935	*hanA*	CRC_01160	92.47	CRD_00595	90.32	Heterocyst differentiation [13]
alr0093	*hcwA*	-	-	-	-	Heterocyst polysaccharide and envelope formation [4]
alr1086	*henR*	CRC_00384	69.04	CRD_03033	68.51	
alr2835	*hepA*†	CRC_01570	57.19	CRD_02099	56.09	
alr3698	*hepB*	CRC_03468	67.94	CRD_01842	68.46	
alr2834	*hepC*	CRC_01572	41.50	CRD_02101	43.19	
all4496	*hepK*	-	-	-	-	
alr0117	*hepN*	CRC_00406	48.58	CRD_01826	47.33	
all2760	*hepS*	-	-	-	-	

alr2817	*hetC*	-	-	-	-	Required for pro-heterocyst cell division cessation[14]
alr3546	*hetF*	CRC_02207	52.46	CRD_00315	58.04	Heterocyst differentiation [15,16]
all3740	*hetL*	-	-	-	-	
alr5357	*hetM*† *(hglB)*	CRC_02052	65.60	-	-	Heterocyst glycolipid-layer formation [17]
alr5358	*hetN*	-	-	-	-	Late stage heterocyst pattern maintenance [18]
alr2818	*hetP*	CRC_01587	58.11	-	-	Heterocyst differentiation and maturation[19-22]
alr3234	*hetP-like*	CRC_00980	69.33	CRD_00495	69.33	
alr3234	*hetP-like*	CRC_02972	41.26	-	-	
alr2902	*hetP-like*	CRC_01674	61.19	CRD_02196	62.68	
alr2339	*hetR*†	CRC_03184	83.94	CRD_01519	82.94	
alr2300	*hetY*	CRC_02119	65.94	CRD_02372	64.74	
alr0099	*hetZ*	CRC_02801	79.07	CRD_02292	79.69	
all5359	*hetI*	CRC_01556	51.96	CRD_01379	53.27	Heterocyst glycolipid-layer formation[23-26]
all5345	*hgdA*	CRC_02043	69.23	-	-	
all5347	*hgdB*	CRC_02045	63.93	CRD_00126	48	
all5346	*hgdC*	CRC_02044	82.39	CRD_00127	61.47	
alr5356	*hglA*	CRC_02051	81.40	CRD_00121	75.62	
alr5355	*hglC*	CRC_02050	61.19	-	-	
alr5354	*hglD*	CRC_02049	71.49	-	-	
alr5351	*hgl*E_A	CRC_02047	65.96	-	-	
all1646	*hgl*E_2	-	-	-	-	
alr5352	*hgl*F	-	-	-	-	
alr5353	*hgl*G	CRC_02048	64	-	-	
all0813	*hgl*K	CRC_00692	54.93	CRD_01636	54.24	
asr4517	*nbl*A	CRC_02186	82.25	CRD_00293	80.64	Phycobiliprotein degradation [27]
all4312	*nrr*A	CRC_00297	86.43	CRD_01945	85.27	Transcriptional regulator of *hetR* [28]
alr4392	*ntcA*†	CRC_00858	96.80	CRD_00550	91.94	N_2 metabolism and heterocyst differentiation regulators [29,30]
all0602	*ntcB*	CRC_01749	77.59	CRD_01496	77.27	
all0521	*patA*†	CRC_01956	40.15	-	-	Heterocyst differentiation and initial pattern formation
all2512	*patB*	CRC_01763	73.48	-	-	

alr4812	*patN*	CRC_03501	60.64	CRD_01874	61.11	[22,31-34]
asl2301	*patS*	CRC_02157	29*	CRD_02133	23*	
asr0101	*patU3*	CRC_02800	51.76	CRD_02293	56.20	
alr2502	*pkn22*	-	-	-	-	NtcA mediated iron metabolism regulation [35,36]
alr0709	*pkn41*	-	-	-	-	
alr0710	*pkn42*	-	-	-	-	
alr4366	*pknA*	-	-	-	-	Heterocyst differentiation [37]
alr4368	*pknD*	CRC_02958	50.49	CRD_00759	47	N_2 regulation [38]
alr3732	*pknE*	-	-	-	-	Heterocyst maturation [38,39]
alr3731	*prpA*	-	-	-	-	
all1731	*prpJ*	CRC_01339	55.29	CRD_02900	53.52	
alr4516	*prpS*	CRC_02187	68.46	CRD_00294	61.95	Modification of PII (GlnB) [12]
all1692	*sigC*	CRC_01434	79.21	CRD_00657	78.60	Transcriptional Regulators [40]
alr3800	*sigE*	CRC_02165	83.48	CRD_00081	84.40	
alr3280	*sigG*	CRC_00607	87.09	CRD_02710	86.23	
asr1734	NA	CRC_01342	87.09	CRD_02898	84.94	Heterocyst development inhibitor [41]
all5343	NA	CRC_02041	55.84	-	-	Heterocyst glycolipid-layer formation [23]
alr5348	NA	-	-	-	-	
asr5349	NA	-	-	-	-	
alr2825	NA	CRC_01582	89.10	CRD_02110	89.10	Heterocyst polysaccharide envelope formation [42]
alr2827	NA	CRC_01580	87.64	CRD_02108	86.09	
alr2831	NA	CRC_01575	79.44	CRD_02104	79.21	
alr2833	NA	CRC_01573	70.88	CRD_02102	70.08	
alr2837	NA	CRC_01568	54.76	CRD_02097	54.76	
alr2839	NA	CRC_01567	62.46	CRD_02096	62.72	
alr2841	NA	CRC_01565	49.77	CRD_02093	49.51	

* To assign the corresponding CDS as a *patS* homologue other features than amino-acid sequence identity were considered (see text). NA, not asinged.

† Expression confirmed by qPCR.

1. Koksharova OA, Wolk CP (2002) Novel DNA-Binding Proteins in the Cyanobacterium *Anabaena* sp. Strain PCC 7120. J Bacteriol 184: 3931-3940.

2. Zhao YH, Shi YM, Zhao WX, Huang X, Wang DH, et al. (2005) CcbP, a calcium-binding protein from *Anabaena* sp PCC 7120 provides evidence that calcium ions regulate heterocyst differentiation. Proc Natl Acad Sci U S A 102: 5744-5748.

3. Frias JE, Herrero A, Flores E (2003) Open Reading Frame all0601 from *Anabaena* sp. Strain PCC 7120 Represents a Novel Gene, *cnaT*, Required for Expression of the Nitrate Assimilation nir Operon. J Bacteriol 185: 5037-5044.

4. Fan Q, Lechno-Yossef S, Ehira S, Kaneko T, Ohmori M, et al. (2006) Signal Transduction Genes Required for Heterocyst Maturation in *Anabaena* sp. Strain PCC 7120. J Bacteriol 188: 6688-6693.

5. Cai Y, Wolk CP (1997) *Anabaena* sp. strain PCC 7120 responds to nitrogen deprivation with a cascade-like sequence of transcriptional activations. J Bacteriol 179: 267-271.

6. Fiedler G, Arnold M, Hannus S, Maldener I (1998) The DevBCA exporter is essential for envelope formation in heterocysts of the cyanobacterium *Anabaena* sp. strain PCC 7120. Mol Microbiol 27: 1193-1202.

7. Ramirez ME, Hebbar PB, Zhou RB, Wolk CP, Curtis SE (2005) *Anabaena* sp strain PCC 7120 gene *devH* is required for synthesis of the heterocyst glycolipid layer. J Bacteriol 187: 2326-2331.

8. Campbell EL, Hagen KD, Cohen MF, Summers ML, Meeks JC (1996) The *devR* gene product is characteristic of receivers of two-component regulatory systems and is essential for heterocyst development in the filamentous Cyanobacterium *Nostoc* sp strain ATCC 29133. J Bacteriol 178: 2037-2043.

9. Schrautemeier B, Böhme H (1985) A distinct ferredoxin for nitrogen fixation isolated from heterocysts of the cyanobacterium *Anabaena variabilis*. FEBS Letters 184: 304-308.

10. Sakr S, Jeanjean R, Zhang CC, Arcondeguy T (2006) Inhibition of cell division suppresses heterocyst development in *Anabaena* sp strain PCC 7120. J Bacteriol 188: 1396-1404.

11. Lopez-Gomollon S, Hernandez JA, Wolk CP, Peleato ML, Fillat MF (2007) Expression of *furA* is modulated by NtcA and strongly enhanced in heterocysts of *Anabaena* sp PCC 7120. Microbiology-Sgm 153: 42-50.

12. Laurent S, Forchhammer K, Gonzalez L, Heulin T, Zhang CC, et al. (2004) Cell-type specific modification of PII is involved in the regulation of nitrogen metabolism in the cyanobacterium *Anabaena* PCC 7120. FEBS Lett 576: 261-265.

13. Khudyakov I, Wolk CP (1996) Evidence that the *hanA* gene coding for HU protein is essential for heterocyst differentiation in, and cyanophage A-4(L) sensitivity of, *Anabaena* sp strain PCC 7120. J Bacteriol 178: 3572-3577.

14. Xu X, Wolk CP (2001) Role for *hetC* in the transition to a nondividing state during heterocyst differentiation in *Anabaena* sp. J Bacteriol 183: 393-396.

15. Wong FCY, Meeks JC (2001) The *hetF* gene product is essential to heterocyst differentiation and affects HetR function in the cyanobacterium *Nostoc punctiforme*. J Bacteriol 183: 2654-2661.

16. Liu DA, Golden JW (2002) *hetL* overexpression stimulates heterocyst formation in *Anabaena* sp. strain PCC 7120. J Bacteriol 184: 6873-6881.

17. Maldener I, Hannus S, Kammerer M (2003) Description of five mutants of the cyanobacterium *Anabaena* sp strain PCC 7120 affected in heterocyst differentiation and identification of the transposon-tagged genes. FEMS Microbiol Lett 224: 205-213.

18. Callahan SM, Buikema WJ (2001) The role of HetN in maintenance of the heterocyst pattern in *Anabaena* sp. PCC 7120. Mol Microbiol 40: 941-950.

19. Fernandez-Pinas F, Leganes F, Wolk CP (1994) A third genetic locus required for the formation of heterocysts in *Anabaena* sp. strain PCC 7120. J Bacteriol 176: 5277-5283.

20. Buikema WJ, Haselkorn R (1991) Characterization of a gene controlling heterocyst differentiation in the cyanobacterium *Anabaena* 7120. Genes Dev 5: 321-330.

21. Yoon HS, Lee MH, Xiong J, Golden JW (2003) *Anabaena* sp strain PCC 7120 *hetY* gene influences heterocyst development. J Bacteriol 185: 6995-7000.

22. Zhang W, Du Y, Khudyakov I, Fan Q, Gao H, et al. (2007) A gene cluster that regulates both heterocyst differentiation and pattern formation in *Anabaena* sp strain PCC 7120. Mol Microbiol 66: 1429-1443.

23. Fan Q, Huang G, Lechno-Yossef S, Wolk CP, Kaneko T, et al. (2005) Clustered genes required for synthesis and deposition of envelope glycolipids in *Anabaena* sp. strain PCC 7120. Mol Microbiol 58: 227-243.

24. Black K, Buikema WJ, Haselkorn R (1995) The *hglK* gene is required for localization of heterocyst-specific glycolipids in the cyanobacterium *Anabaena* sp. strain PCC 7120. J Bacteriol 177: 6440-6448.

25. Bauer CC, Ramaswamy KS, Endley S, Scappino LA, Golden JW, et al. (1997) Suppression of heterocyst differentiation in *Anabaena* PCC 7120 by a cosmid carrying wild-type genes encoding enzymes for fatty acid synthesis. FEMS Microbiol Lett 151: 23-30.

26. Campbell EL, Cohen MF, Meeks JC (1997) A polyketide-synthase-like gene is involved in the synthesis of heterocyst glycolipids in *Nostoc punctiforme* strain ATCC 29133. Arch Microbiol 167: 251-258.

27. Baier K, Lehmann H, Stephan DP, Lockau W (2004) NblA is essential for phycobilisome degradation in *Anabaena* sp strain PCC 7120 but not for development of functional heterocysts. Microbiology-Sgm 150: 2739-2749.

28. Ehira S, Ohmori M (2006) NrrA, a nitrogen-responsive response regulator facilitates heterocyst development in the cyanobacterium *Anabaena* sp strain PCC 7120. Mol Microbiol 59: 1692-1703.

29. Wei TF, Ramasubramanian TS, Golden JW (1994) *Anabaena* sp. strain PCC 7120 *ntcA* gene required for growth on nitrate and heterocyst development. J Bacteriol 176: 4473-4482.

30. Frias JE, Flores E, Herrero A (2000) Activation of the *Anabaena nir* operon promoter requires both NtcA (CAP family) and NtcB (LysR family) transcription factors. Mol Microbiol 38: 613-625.

31. Liang J, Scappino L, Haselkorn R (1992) The *patA* gene-product, which contains a region similar to CheY of *Escherichia coli*, controls heterocyst pattern-formation in the cyanobacterium *Anabaena* 7120. Proc Natl Acad Sci U S A 89: 5655-5659.

32. Jones KM, Buikema WJ, Haselkorn R (2003) Heterocyst-Specific Expression of *patB*, a Gene Required for Nitrogen Fixation in *Anabaena* sp. Strain PCC 7120. J Bacteriol 185: 2306-2314.

33. Meeks JC, Elhai J (2002) Regulation of cellular differentiation in filamentous cyanobacteria in free-living and plant-associated symbiotic growth states. Microbiol Mol Biol Rev 66: 94-121.

34. Yoon HS, Golden JW (1998) Heterocyst pattern formation controlled by a diffusible peptide. Science 282: 935-938.

35. Cheng Y, Li JH, Shi L, Wang L, Latifi A, et al. (2006) A pair of iron-responsive genes encoding protein kinases with a Ser/Thr kinase domain and a His kinase domain are regulated by NtcA in the cyanobacterium *Anabaena* sp strain PCC 7120. J Bacteriol 188: 4822-4829.

36. Xu WL, Jeanjean R, Liu YD, Zhang CC (2003) *pkn22* (alr2502) encoding a putative Ser/Thr kinase in the cyanobacterium *Anabaena* sp PCC 7120 is induced by both iron starvation and oxidative stress and regulates the expression of *isiA*. FEBS Lett 553: 179-182.

37. Zhang CC (1993) A gene encoding a protein related to eukaryotic protein kinases from the filamentous heterocystous cyanobacterium *Anabaena* PCC 7120. Proc Natl Acad Sci U S A 90: 11840-11844.

38. Zhang CC, Libs L (1998) Cloning and characterisation of the *pknD* gene encoding an eukaryotic-type protein kinase in the cyanobacterium *Anabaena* sp. PCC7120. Mol Gen Genet 258: 26-33.

39. Jang JC, Wang L, Jeanjean R, Zhang CC (2007) PrpJ, a PP2C-type protein phosphatase located on the plasma membrane, is involved in heterocyst maturation in the cyanobacterium *Anabaena* sp PCC 7120. Mol Microbiol 64: 347-358.

40. Aldea MR, Mella-Herrera RA, Golden JW (2007) Sigma Factor Genes *sigC*, *sigE*, and *sigG* are Upregulated in Heterocysts of the Cyanobacterium *Anabaena* sp. Strain PCC 7120. J Bacteriol 189: 8392-8396.

41. Wu XQ, Lee DW, Mella RA, Golden JW (2007) The *Anabaena* sp strain PCC 7120 asr1734 gene encodes a negative regulator of heterocyst development. Mol Microbiol 64: 782-794.

42. Huang G, Fan Q, Lechno-Yossef S, Wojciuch E, Wolk CP, et al. (2005) Clustered genes required for the synthesis of heterocyst envelope polysaccharide in *Anabaena* sp. strain PCC 7120. J Bacteriol 187: 1114-1123.
