# Supplementary material for: The Smallest Known Genomes of Multicellular and Toxic Cyanobacteria: Comparison, Minimal Gene Sets for Linked Traits and the Evolutionary Implications
Source: PLoS One. 2010 Feb 16;5(2):e9235. doi: 10.1371/journal.pone.0009235 (PMC2821919; doi:10.1371/journal.pone.0009235)
Supplement: Methods S1 — Supplementary Material and Methods (0.06 MB DOC) [file pone.0009235.s012.doc]

**RFLP by PFGE and genome size estimation**

Intact chromosomal DNA from strains CS-505 and D9 embedded into agarose plugs was digested with the high frequency cut restriction enzyme *Mlu* I as described previously [1]. The estimation of the genomic sizes was achieved using as standard, genomic DNA from *Vibrio parahaemolyticus* RIMD 2210633 (Vpkx) digested with *Not* I [2]. The genome sequence of Vpkx is available and comprises two chromosomes of 3.3 and 1.8 Mb. A calibration curve (*r*^2^ = 0.99012) was generated assuming a logarithmic migration of the DNA fragments during the electrophoresis. Bands showing higher intensity in CS-505 and D9 were considered as double.

**RNA extraction, cDNA synthesis and qPCR detection of genes involved in N-metabolism and heterocyst pattern formation**

Total RNA was extracted from mid-exponential growth cultures using Rneasy mini kit (Qiagen, Hilden, Germany) following the manufacturer’s instructions. Briefly, frozen samples were thawed on ice, and approximately two small spatulas full of 0.1 mm diameter glass beads were added to the sample. The cells were sheared for 2 X 120 s using a Qiagen Bead Beater (Hilden, Germany). The supernatant was separated from the glass beads and cell debris by centrifugation (10 min, 13,200 rpm, 4°C). A DNA digestion was performed after the RNA isolation for 1 h at RT and a final clean up with a second DNA on tube digestion was performed to remove any possible DNA remaining on the RNA samples. RNA was quantified using the NanoDrop ND- 1000 Spectrophotometer (NanoDrop Technologies, Wilmington, USA) and the RNA purity was assessed using the ratio A_260_/A_230_. If required, samples were further cleaned using the Microcon Elute System (Millipore, Massachusetts, USA). RNA integrity was checked on RNA nano chips using the Agilent Bioanalyzer 2100 (Agilent, Santa Clara, CA, USA).

cDNA was synthesized from 500 ng total RNA using random hexamers with the Omniscript RT kit from Qiagen. All primers were designed using the Primer Express 3.0 (Applied Biosystems, Darmstadt, Germany) software and synthesised from MWG Biotechnologies (Eurofins MWG Operon, Ebersberg, Germany). Primer sequence are as follows: q505nifHF 5’ GCTGCTGAGAAAGGTGCTGTAG 3`; q505nifHR 5’ GGAATCCGGCCAGCATTACT 3’; q505fdxHF 5’ CAGCAGCAGAAGCAGATATTGAA 3’; q505fdxHR 5’CCACACAACTAGAGCAAGAACCA3’; q505nifBF 5’ TCAACCAGGTCCGACTAATAAAGAA 3’; q505nifBR 5’ CGCATCTGCTCGACACTGA 3’; q505nifVF 5’ TTGGAACCGAGCAGTTATTTCA 3’; q505nifVR 5’ GCTAGCGATTTGGATTCCAGAA 3’; qhetRF 5’ AGTTGCCACAGCAGCATCAA 3’; qhetRR 5’ GGAGAGTCAATCCGGGTAACC 3’; qntcAF 5’ TTTTACTGCGGTGGAATTGCT 3’; qntcAR 5’ TTCCTTGAGGGCCTGCTCTAC 3’; q505patAF 5’ AACGTCAATTCCTTGGTGCAA 3’; q505patAR 5’ AGACCAGATTCTTCTCGGGAAAC 3’; qhepAF 5’ TGGTGCATCAGGTGGTGGTA 3’; qhepAR 5’ CCCGTTAGTTGGTTCATGGAA 3’; q505hetMF 5’ TAGGCGCGGAAGCAGTTCT 3’; q505hetMR 5’ CCAGTTGCTCCGGTTATCAAG 3’.

The expression of nitrogen metabolism genes was tested in CS-505 and D9 (table below). A 20 µl qPCR reaction was composed of 1 µl of a 10-fold diluted cDNA, forward and reverse primers at a concentration of 100 nM, and 10 µl 2x SYBR Green PCR Master Mix (Applied Biosystems, Darmstadt, Germany). Pure RNA was used to test for the presence of genomic DNA in the RNA samples. Cycle parameters were as follows: initial denaturation at 95°C/10 min, followed by 40 cycles of 95°C/15 sec and 59°C/1 min. Finally a product-primer dissociation step was utilized to verify formation of a single unique product/primer dimerization.

| gene | Efficiency (%) | slope | r^2^ | Ct CS-505 | Ct D9 |
| --- | --- | --- | --- | --- | --- |
| *nifH* | 93,8 | -3,11 | 0,9934 | 22,30 | ND |
| *fdxH* | NT | NT | NT | 26,23 | ND |
| *nifB* | NT | NT | NT | 25,22 | ND |
| *nifV* | NT | NT | NT | 25,29 | ND |
| *hetR* | 104 | -3,46 | 0,9996 | 21,45 | 14,11 |
| *ntcA* | 102,7 | -3,41 | 0,9986 | 21,38 | 15,01 |
| *patA* | 89,8 | -2,99 | 0,9918 | 20,77 | ND |
| *hepA* | NT | NT | NT | 26,04 | 14,40 |
| *hetM* | NT | NT | NT | 21,38 | ND |

NT: Not tested. ND: Not detected

**Genomic DNA isolation and PCR amplification of the hydrogenase genes *hupC* and *hypF***

DNA was extracted using the CTAB method [3]. The following PCR primers targeting the region upstream and downstream the CYN gene cluster were used: HYPa 5-GGGGTGGACAGTGGTCATAC-3, HUPa 5-TGGGTGTTCCTCATCAACAA-3 forward and reverse repectively, and targeting the region between the maturation hydrogenase genes *hypF* and *hupC* of D9: HYPb 5-CCTCCAAACGATGGAGGAAT-3, HUPb 5-GGGTGTTCCTCATCCACAAT-3 forward and reverse, respectively (Figure 4). The PCR reaction contained 50–100 ng of genomic DNA, the reagents for each 30 µl amplification reaction were: 0.25 U Taq DNA polymerase (Invitrogen, California, USA); 3 µl 10X PCR buffer; 2.5mM MgCl; 0.4 mM primers; and 0.93 mM of each deoxynucleoside triphosphate (Promega, Wisconsin, USA). Thermal cycling was performed in an Eppendorf Mastercycler, under the following conditions: initial DNA denaturation at 99ºC/1 min, 30 cycles 94ºC/15 sec, 53ºC/1 min, 72ºC/1.5 min and a final extension at 72ºC/7 min. All PCR products were verified by gel electrophoresis (1% agarose) and visualized under UV transilumination after staining with ethidium bromide.

1. Stucken K, Murillo AA, Soto-Liebe K, Fuentes-Valdés JJ, Méndez MA, et al. (2009) Toxicity phenotype does not correlate with phylogeny of *Cylindrospermopsis raciborskii* strains. Syst Appl Microbiol 32: 37-48.

2. Hara-Kudo Y, Sugiyama K, Nishibuchi M, Chowdhury A, Yatsuyanagi J, et al. (2003) Prevalence of Pandemic Thermostable Direct Hemolysin-Producing Vibrio parahaemolyticus O3:K6 in Seafood and the Coastal Environment in Japan. Appl Environ Microbiol 69: 3883-3891.

3. Ausubel FM, Brent R, Kinston R, Moore D, Seidman JG, et al. (1992) Current protocols in molecular biology. New York: Greene Publishing Associates and Wiley-Inter-science. pp. 2.1.1–2.4.5.
